# Early afterdepolarizations promote transmural reentry in ischemic human ventricles with reduced repolarization reserve

**DOI:** 10.1016/j.pbiomolbio.2016.01.008

**Published:** 2016-01

**Authors:** Sara Dutta, Ana Mincholé, Ernesto Zacur, T. Alexander Quinn, Peter Taggart, Blanca Rodriguez

**Affiliations:** a Department of Computer Science, BHF Centre of Research Excellence, University of Oxford, Oxford, United Kingdom; b Institute of Biomedical Engineering, University of Oxford, Oxford, United Kingdom; c Department of Physiology and Biophysics, Dalhousie University, Canada; d Institute of Cardiovascular Science, University College London, Bars Heart Centre, United Kingdom

**Keywords:** Ischemic heart disease, Computer-based model, Ventricular arrhythmia, Potassium channels, Repolarization

## Abstract

**Aims:**

Acute ischemia is a major cause of sudden arrhythmic death, further promoted by potassium current blockers. Macro-reentry around the ischemic region and early afterdepolarizations (EADs) caused by electrotonic current have been suggested as potential mechanisms in animal and isolated cell studies. However, ventricular and human-specific arrhythmia mechanisms and their modulation by repolarization reserve remain unclear. The goal of this paper is to unravel multiscale mechanisms underlying the modulation of arrhythmic risk by potassium current (I_Kr_) block in human ventricles with acute regional ischemia.

**Methods and results:**

A human ventricular biophysically-detailed model, with acute regional ischemia is constructed by integrating experimental knowledge on the electrophysiological ionic alterations caused by coronary occlusion. Arrhythmic risk is evaluated by determining the vulnerable window (VW) for reentry following ectopy at the ischemic border zone. Macro-reentry around the ischemic region is the main reentrant mechanism in the ischemic human ventricle with increased repolarization reserve due to the ATP-sensitive potassium current (I_K(ATP)_) activation. Prolongation of refractoriness by 4% caused by 30% I_Kr_ reduction counteracts the establishment of macro-reentry and reduces the VW for reentry (by 23.5%). However, a further decrease in repolarization reserve (50% I_Kr_ reduction) is less anti-arrhythmic despite further prolongation of refractoriness. This is due to the establishment of transmural reentry enabled by electrotonically-triggered EADs in the ischemic border zone. EADs are produced by L-type calcium current (I_CaL_) reactivation due to prolonged low amplitude electrotonic current injected during the repolarization phase.

**Conclusions:**

Electrotonically-triggered EADs are identified as a potential mechanism facilitating intramural reentry in a regionally-ischemic human ventricles model with reduced repolarization reserve.

## 1 Introduction

Acute ischemia associated with coronary heart disease is a leading cause of sudden arrhythmic death. Pro-arrhythmic mechanisms of acute ischemia have been extensively investigated, although often in animal models at the single cell or tissue level rather than in human ventricles. Seminal studies by Janse et al. in pig and dog hearts highlight the complexity of the pro-arrhythmic and spatio-temporally dynamic substrate in acute ischemia (Janse and Wit, 1989, Janse et al., 1980, Janse et al., 1979). Heterogeneity in excitability and repolarization properties across the border between the normal and ischemic areas leads to the establishment of reentry around the ischemic region following ectopic excitation. The same studies also showed intramural reentry in certain cases (highlighting the potential variability in the mechanisms), recorded with microelectrodes on and 4 and 8 mm below the epicardial surface in pig hearts affected by left anterior descending coronary artery occlusion. However, the mechanisms that determine reentry formation and intramural patterns in acute ischemia in the three-dimensional human heart remain unclear, due to low resolution of intramural recordings.

Electrotonic current flowing due to gradients in membrane potential in heterogeneous tissue has been hypothesized as a potentially-important pro-arrhythmic factor in acute ischemia (Coronel et al., 1991, Janse et al., 1980). One potential mechanism is that electrotonic current flow could promote the occurrence of early afterdepolarizations (EADs) in acute ischemia (Kumar and Joyner, 1994, Verkerk et al., 2000). Single cell studies showed EADs triggered by electrotonic current in human cardiomyocytes from failing hearts, but only when cells in phase 3 repolarization were coupled to very high potentials corresponding to plateau level (such as 0 mV) (Verkerk et al., 2000). Neighboring cells would therefore have to experience very different membrane potential levels for this to occur. Furthermore, electrotonic interactions in well coupled tissue (as is still the case in the early phase of acute ischemia) are known to decrease the propensity of EAD formation in tissue (Weiss et al., 2010, Xie et al., 2010), even if they can be triggered in single cells as in Verkerk et al. (2000). Therefore, it remains unclear what conditions are needed for EADs to be triggered by electrotonic current, as well as the role of EAD formation in the regionally ischemic human heart.

Pro-arrhythmic mechanisms including both reentry and EAD formation strongly depend on the balance of currents during repolarization, and the specific degree of redundancy in repolarizing currents, termed repolarization reserve, available. This is affected by a variety of causes including inter-subject differences in ion channel density, drug effects and mutations. Individuals with reduced repolarization reserve are known to be at higher arrhythmic risk, particularly in the presence of a pro-arrhythmic substrate such as ischemia due to coronary heart disease as shown in clinical studies (Steinberg et al., 2014, Waldo et al., 1996). Furthermore, clinical studies show that interventions that reduce repolarizing currents such as class III drugs result in increased risk of longer-term hospitalization and death in patients with coronary heart disease (Steinberg et al., 2014). Studies, such as the Survival With ORal D-Sotalol (SWORD) trial, also demonstrate that administration of the hERG (human Ether-à-go-go Related Gene) blocker sotalol is associated with increased mortality in patients suffering from recent myocardial infarction, related to ischemic heart disease (Waldo et al., 1996). How changes in the degree of potassium current available during repolarization alters the ischemic human heart to increase arrhythmic risk remains unclear.

Investigating the pro-arrhythmic substrate of acute ischemia and its alteration by potassium block experimentally is very challenging. Of particular difficulty is mapping the three-dimensional volume of the heart with enough spatio-temporal resolution to investigate complex and irregular intramural propagation patterns through the ventricular wall. Microelectrode recordings and optical mapping have both been used in animal studies and they both suffer from a different set of limitations. The former exhibit low resolution, whereas the latter provides recordings only of the tissue surface and with photon scattering averaging effects (Bishop et al., 2007, p. 2007). Acute ischemia is further complicated by the fact that the electrophysiological changes induced are dynamic in time and reproducibility of experimental findings is compromised.

In this study, we investigate the mechanisms of increased arrhythmic risk in the heterogeneous substrate caused by acute myocardial ischemia for different degrees of reduced repolarization reserve using an anatomically-based biophysically-detailed human ventricles model. We hypothesize that in human ventricles with low repolarization reserve, gradients in membrane potential across the ischemic border zone promote the occurrence of electrotonically-triggered EADs that contribute to the establishment of transmural micro-reentry. By analyzing high spatio-resolution simulation data, we unravel the three-dimensional mechanisms of EAD formation and propagation dynamics in the acutely-ischemic human ventricles, their modulation by repolarization reserve and their potential contribution to increased arrhythmic risk.

## 2 Methods

### 2.1 Human ventricular model in acute regional ischemia

An anatomically-based multiscale model of regionally-ischemic human ventricles was developed based on extensive experimental recordings from (Carmeliet, 1999, Coronel et al., 1991, Coronel et al., 1988, Durrer et al., 1970, Edvardsson et al., 1980, Glukhov et al., 2010, Lee et al., 1981, Spector et al., 1996, Sutton et al., 2000, Taggart et al., 2001, Wilensky et al., 1986), including biophysically-detailed membrane kinetics, ischemia induced heterogeneities and fiber orientation (based on the Streeter method (Streeter et al., 1969)). Human ventricle membrane kinetics were simulated with the ten Tusscher 2006 action potential (AP) model (TP06) (Tusscher and Panfilov, 2006) with an added ATP-sensitive potassium current (I_K(ATP)_) (Michailova et al., 2005). The TP06 has been extensively used and shown to be suitable to simulate reentrant arrhythmias as well as ischemic electrophysiological alterations in human (Dutta et al., 2013, Kazbanov et al., 2014). An expanded version of the methods is available as Supplemental Material.

### 2.2 Heterogeneous electrophysiological substrate in the ischemic region

**Fig. 1:**
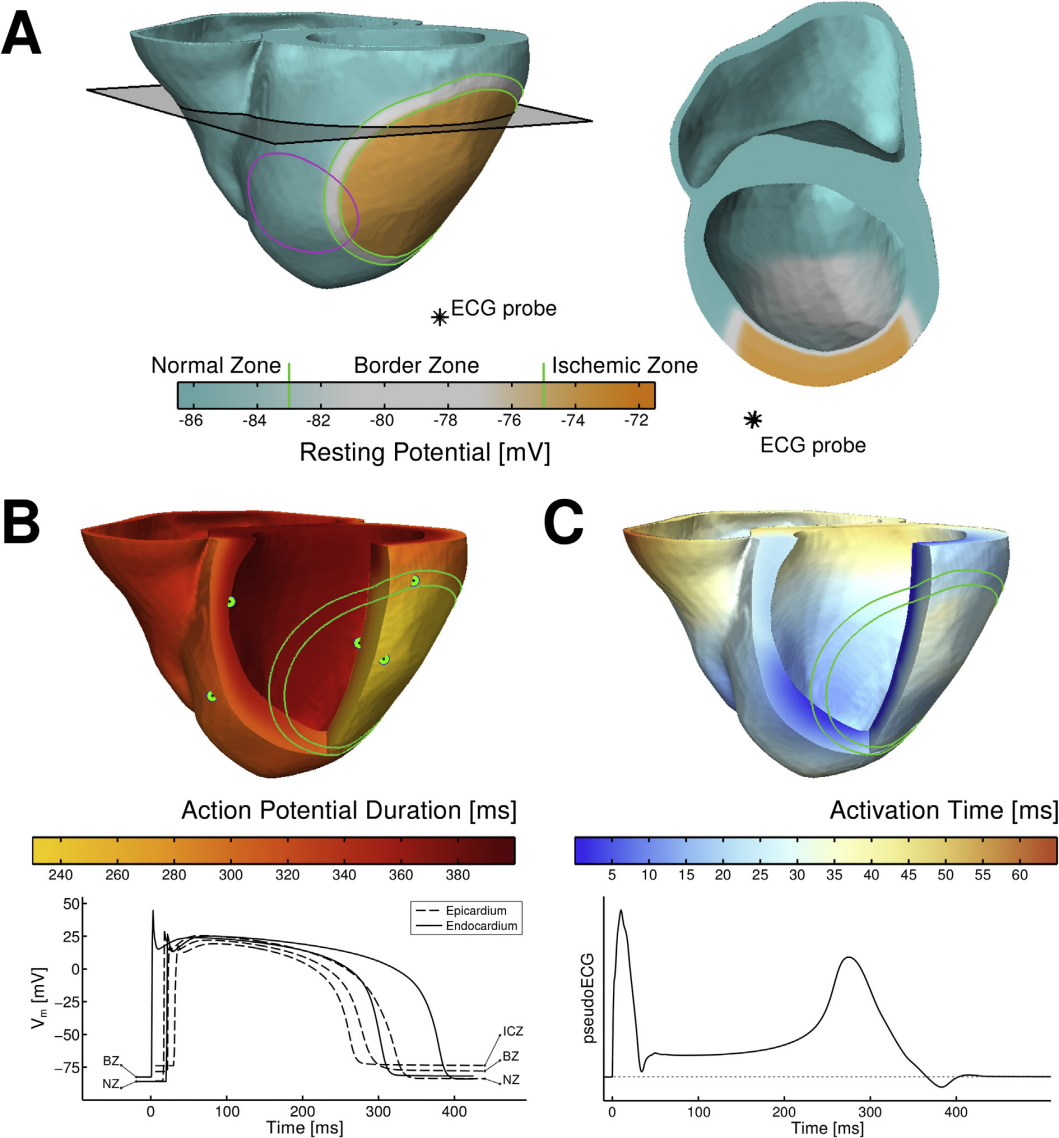
Electrophysiological heterogeneity in the anatomically-based model of the human ventricles in acute regional ischemia. A. Resting potential distribution with elevated potentials in ischemic tissue (green solid line), location of the pseudo-ECG probe (black asterisk) and ectopic stimulation site (magenta solid line). B. Action potential duration (APD) distribution highlighting APD shortening in the ischemic central zone (ICZ) (top) and examples of action potentials in normal zone (NZ), border zone (BZ) and ICZ from the locations marked with green dots in the APD map. C. Activation times distribution (top) and pseudo-ECG (bottom) exhibiting first the QRS complex, followed by a positive T wave (due to the inverse relationship between APD and activation times) and ischemia-induced ST elevation.

Size and location of ischemic regions exhibit great variability. We represented the ischemic region in our model as affecting ∼40% of the left ventricle, which is within the range of 13–72% reported by Lee et al. (1981). (Fig. 1A) and corresponds to greatest likelihood of ventricular fibrillation (Curtis, 1998). The ischemic region was modeled in agreement with experimental findings by Coronel et al., 1991, Rodríguez et al., 2006, Wilensky et al., 1986 including: (i) the ischemic central zone (ICZ); (ii) the border zone (BZ), (iii) and the endocardial BZ (a layer of endocardial tissue with access to oxygen and nutrients from blood in the ventricular cavity) (Coronel et al., 1988, Wilensky et al., 1986).

In the ICZ, ionic alterations caused by acute ischemia were introduced (Coronel et al., 1991, Rodríguez et al., 2006, Wilensky et al., 1986), with a degree of ischemic severity corresponding to highest arrhythmic risk (Carmeliet, 1999, Janse and Wit, 1989, Kazbanov et al., 2014, Rodríguez et al., 2006) during early stages of ischemia (up to 15 min). The BZ included a linear gradient in electrophysiological properties from the ICZ to the normal zone (NZ) tissue as shown in experiments (Coronel et al., 1988, Wilensky et al., 1986). Calcium overload or gap junction changes were not included as they occur at later stages of ischemia (Carmeliet, 1999). The human model also included transmural heterogeneities in AP duration (APD) to reproduce the physiological inverse APD - activation time relationship (Fig. 1B and C) resulting in a positive T wave in the pseudo-ECG, as in data obtained in *in vivo* human hearts (Franz et al., 1987). More details can be found in the Supplemental Material.

### 2.3 Stimulation protocol

Purkinje-like activation was simulated twice with a cycle length (CL) of 800 ms, by stimulating the endocardium (S1) to mimic the activation sequence in Durrer et al. (1970). This was followed by a premature excitation (S2), applied in a region close to the BZ prone to premature beats that mimics findings by Janse et al. (1980). The coupling interval (CI) of the premature stimulus (e.g., time interval difference between S1 and S2) was varied to quantify the vulnerability window (VW) of reentry. The latter was computed as the range of CIs that resulted in reentry in the human ventricles.

As illustrated in Fig. 1, the human ventricles model reproduced the reported electrophysiological heterogeneity caused by regional ischemia, including elevated resting transmembrane potential (V_m_) and short APD (Rodríguez et al., 2006) in the ICZ with respect to NZ, in agreement with *in vivo* human and *ex situ* animal studies (Carmeliet, 1999, Sutton et al., 2000, p. 200; Taggart et al., 2001, Wilensky et al., 1986). Resting potential in the ICZ is −70 mV compared to −86 mV in normal tissue, and APD is 30% shorter in the ICZ than in the NZ, close to the 35% difference reported in human *in vivo* measurements (Sutton et al., 2000). The simulated pseudo-ECG was computed as in Gima and Rudy (2002), as the extracellular unipolar potential at a position 3.6 cm away from the epicardial surface (Fig. 1A), and yielded a physiological QT interval of ∼400 ms and positive T wave, as described in *in vivo* human hearts (Franz et al., 1987).

### 2.4 Reduced repolarization reserve

In order to investigate mechanistic implications of reduced repolarization reserve in acute ischemia, we considered three scenarios represented by a 0, 30 and 50% decrease of the rapidly activating delayed rectifier potassium current (I_Kr_) conductance in the TP06. The simulations yield a 5% and 9% prolongation in QT in the pseudo-ECG, which is similar to the 7% and 12% increase found by Fossa et al. in healthy patients, 2–4 h after being given 160 mg and 320 mg of sotalol respectively (Fossa et al., 2007).

**Fig. 2:**
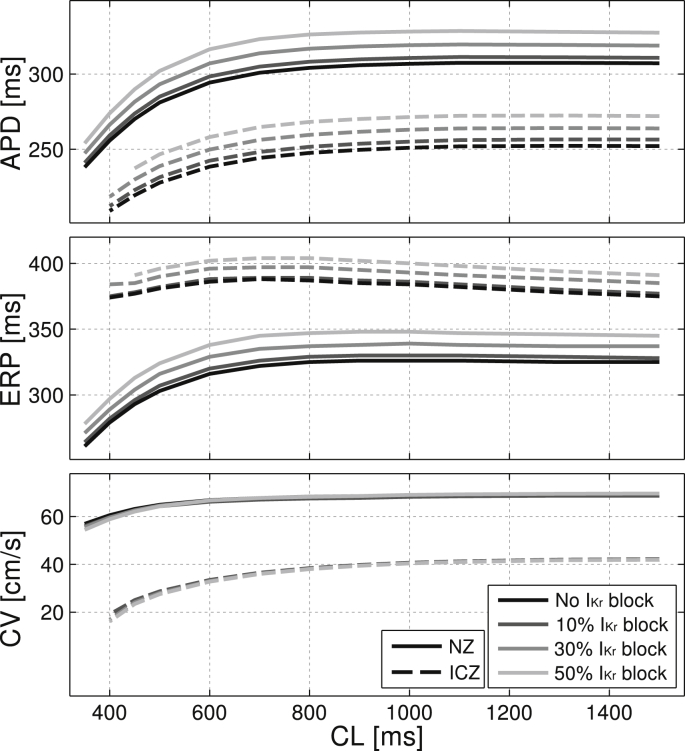
Electrophysiological properties in a one-dimensional homogeneous fiber of human ventricular tissue under control and acute ischemic conditions for varying degrees of the rapidly activating delayed rectifying potassium current (I_Kr_) reduction. Results show steady-state values for APD, effective refractory period (ERP) and conduction velocity (CV) for cycle lengths (CL) ranging from 350 to 1500 ms. Normal and ischemic cells were assigned extracellular potassium concentration ([K^+^]_o_) of 5.4 and 8.5 mmol/L, ATP-sensitive potassium current (I_K(ATP)_) activation of 0 and 5%, and peak conductance of fast sodium current (I_Na_) and L-type calcium current (I_CaL_) of 100 and 75%, respectively, of their original values in the TP06 model.

One-dimensional simulations were conducted to characterize the effect of reduced repolarization reserve in NZ and ICZ tissue for varying degrees of I_Kr_ reduction. APD was calculated at 90% repolarization at steady state for each CL. Effective refractory period (ERP) was evaluated after reaching steady state at each CL and calculated as the shortest CI to induce sustained propagation in the tissue. Conduction velocity (CV) was calculated at steady state from the time interval separating two activation times at two points in the center of the tissue separated by 2 mm. Fig. 2 illustrates the known electrophysiological effects of acute ischemia, including APD shortening, ERP prolongation and slow CV for all degrees of repolarization reserve. Note that no EADs occurred in any of the single cell and one-dimensional simulations presented in Fig. 2.

## 3 Results

### 3.1 Macro-reentrant patterns in the acutely-ischemic human ventricle model support experimental evidence in animal models

**Fig. 3:**
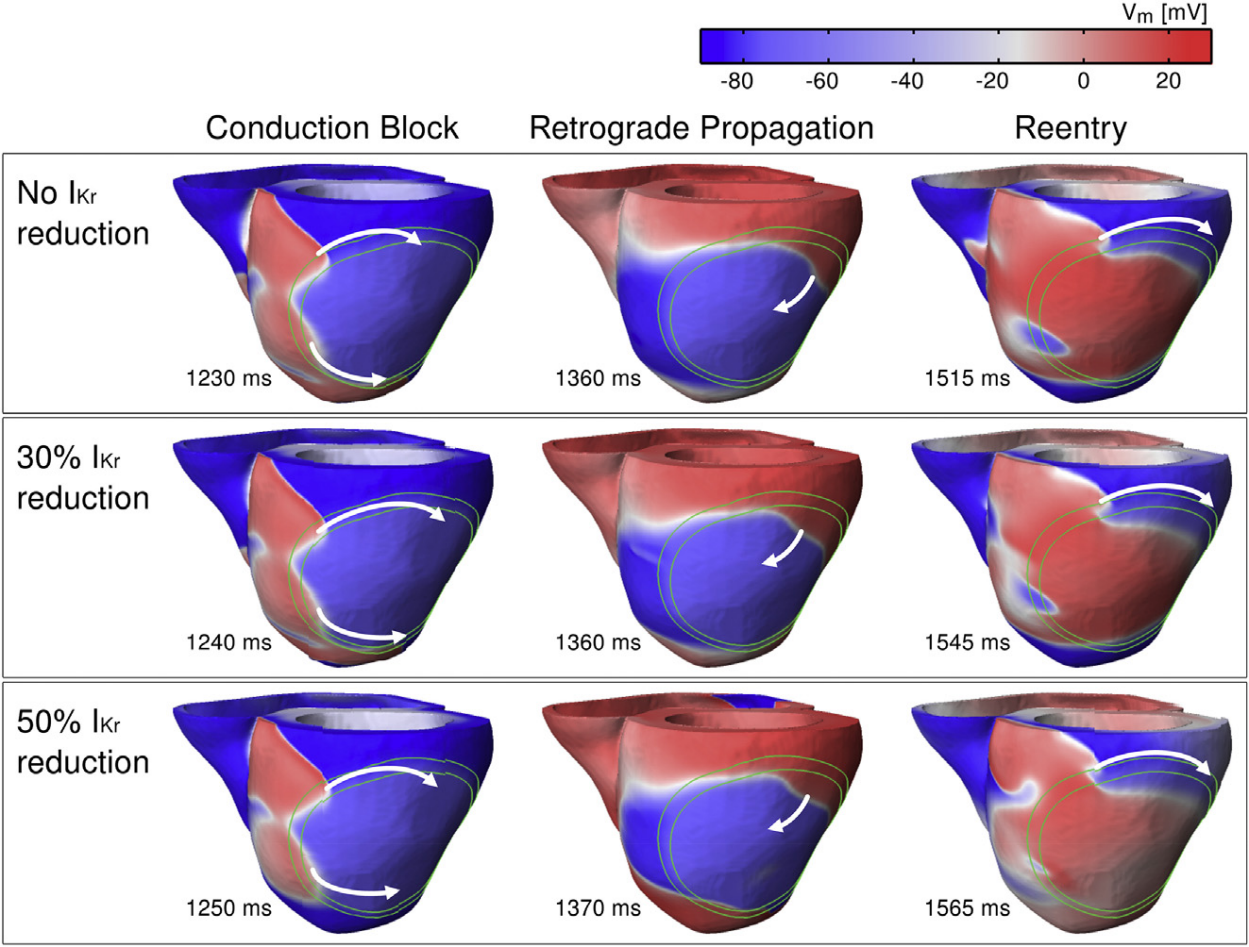
Macro-reentrant pattern of propagation in the human ventricles around the acute ischemic zone in control (top) and for 30% (middle) and 50% (bottom) I_Kr_ reduction following premature excitation applied with coupling intervals (CIs) of 355 ms, 360 and 361 ms, respectively. The limits of the BZ are marked with green lines and the direction of propagation with white arrows.

Simulations show the establishment of macro-reentry around the ischemic region in the human ventricles following premature excitation during the VW for all levels of repolarization reserve. As illustrated in Fig. 3, in all cases, the macro-reentrant pattern was similar, including 3 main phases consistent with experimental patterns (Janse et al., 1980). Firstly, unidirectional conduction block occurs due to prolonged refractoriness in the ICZ, with propagation around this region *via* the NZ and BZ around the ICZ (first column in Fig. 3). Then, retrograde propagation occurs through the ICZ once tissue distal to premature excitation has recovered (middle column in Fig. 3). Finally, wavefront propagation reenters through the NZ (third column in Fig. 3). In all cases, the macro-reentrant pattern was similar and lasted for up to 3 reentry cycles after which it self-terminated. This may indicate that the establishment of sustained ventricular tachycardia and its transition to ventricular fibrillation may require the contribution of more than one ectopic beat and/or pro-arrhythmic substrate factors.

Decreased repolarization reserve by 30% I_Kr_ reduction leads to a decrease of the VW for reentry (65 ms with 30% I_Kr_ reduction vs. 85 ms without I_Kr_ decrease; CI = 355–420 ms for 30% I_Kr_ reduction vs. CI = 335–420 ms for no I_Kr_ decrease). The main anti-arrhythmic mechanism of decreasing I_Kr_ is that prolonged ERP favors conduction block both in the NZ and ICZ at short CIs, therefore leading to either bidirectional block just after premature excitation or failure to sustain retrograde conduction in the ICZ. While the patterns of macro-reentry were similar when I_Kr_ reduction was increased to 50%, the anti-arrhythmic activity of I_Kr_ diminished, as shown by an increased width of the VW (80 ms; CI = 355–435 ms). The causes of this increase in the VW with a further reduction in repolarization reserve are investigated below.

### 3.2 Electrotonically-triggered EADs facilitate transmural reentry near the ischemic border zone under conditions of reduced repolarization reserve

**Fig. 4:**
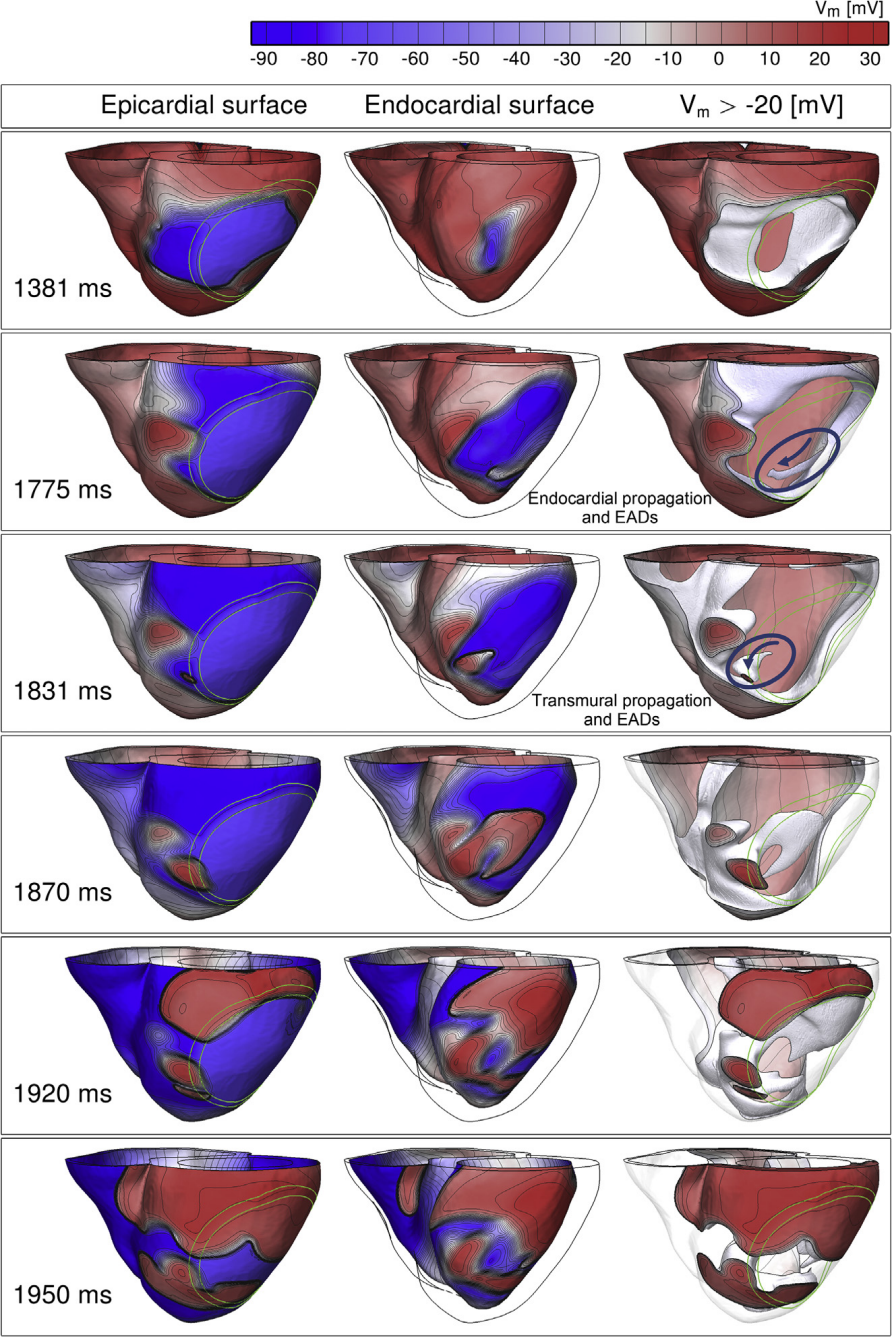
Distribution of transmembrane voltage (V_m_) throughout the ventricles at different times following ectopic excitation resulting initially in macro-reentry, but failure to support retrograde propagation (1381 ms), followed by propagation through the endocardial BZ (1775 ms) and transmurally (1831 ms), leads to intramural reentry (1831–1950 ms). For each time instant, three different views are shown, from left to right: the epicardium, the endocardium and the depolarized tissue with V_m_ above −20 mV (cells with V_m_ < −20 mV are transparent). Isopotential lines are shown in grey linking same potential levels. More details can be found in the supplemental movies.

Detailed analysis of the three-dimensional activation patterns in the simulations revealed that the increase in the VW under conditions of reduced repolarization reserve with 50% I_Kr_ block was caused by transmural reentry supported by electrotonically-triggered EADs near the ischemic BZ, as illustrated in Fig. 4. Firstly, two macro-reentrant cycles are established around the ICZ starting at 1200 ms and 1460 ms (as in Fig. 3). Then, prolonged refractoriness in the ICZ prevents retrograde propagation, and the macro-reentrant propagation dies out (Fig. 4, 1381 ms and 1775 ms panels). However, preserved excitability in the endocardial BZ sustains propagation of a small wavefront (Fig. 4, 1775 ms snapshot, circle and arrow), which finds a nonrefractory tunnel for propagation *via* the lateral BZ, from endocardium to epicardium (Fig. 4, 1831 ms, snapshot, circle and arrow). Following breakthrough on the epicardium, the wavefront reenters back transmurally towards the endocardium and around the ischemic BZ (Fig. 4, 1870 ms, 1920 ms and 1950 ms snapshots).

**Fig. 5:**
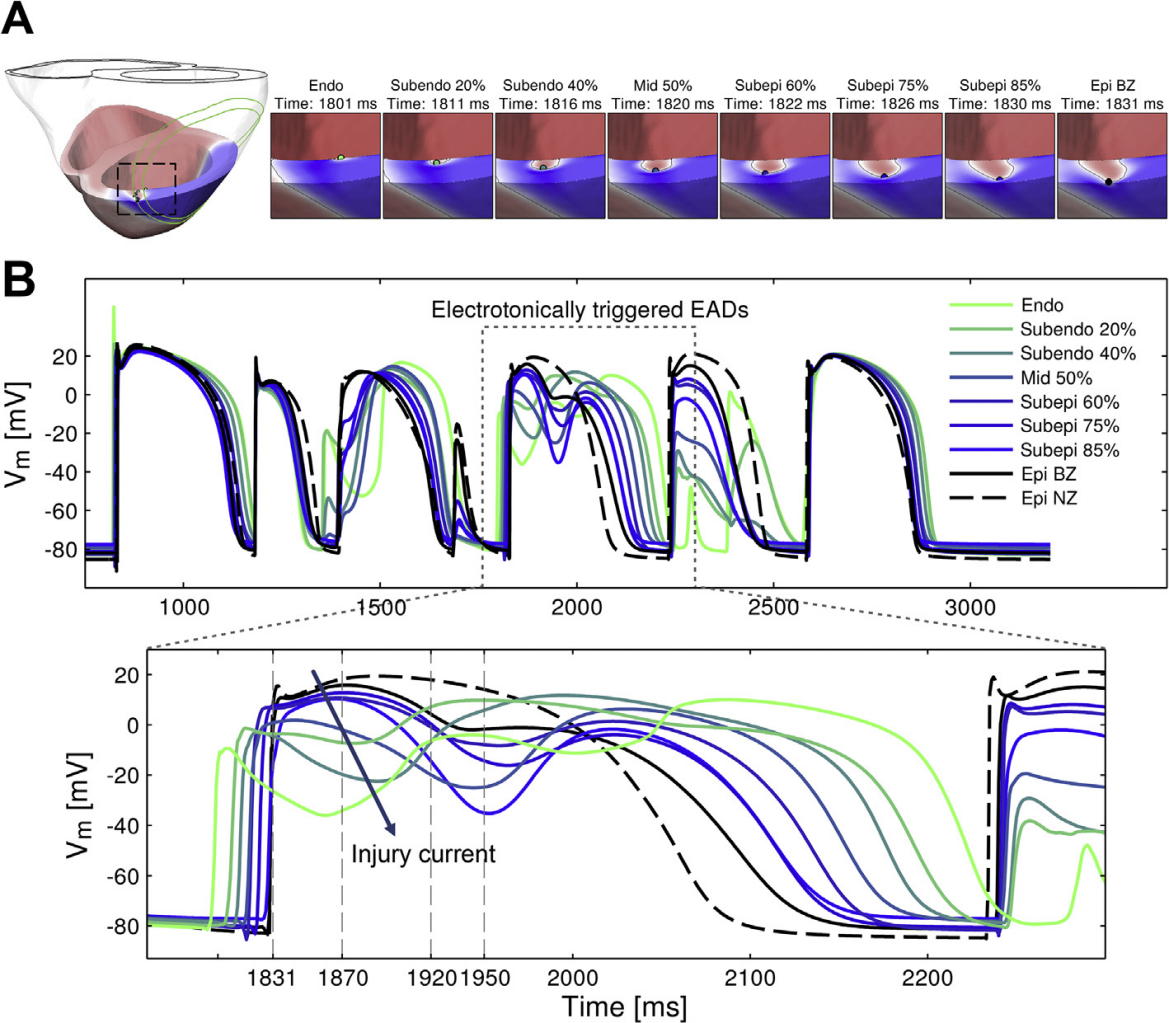
Electrotonically-triggered EADs are recorded near the BZ in the acutely-ischemic human ventricles with 50% I_Kr_ reduction facilitating transmural patterns of reentry. A. Snapshots of a transmural cross-section illustrating propagation of electrical excitation from endocardium to epicardium at the times indicated. B. Time course of V_m_ at the points indicated in panel A from endocardium to epicardium. Legend indicates the transmural location corresponding to each AP trace with % indicating transmural distance from the endocardium. Ectopic excitation is applied at t = 1181 ms (CI = 361 ms as in Fig. 3).

This pattern of transmural propagation is only seen for 50% I_Kr_ block and the cellular-level mechanisms were investigated by analyzing the time course of V_m_ at different locations from endocardium to epicardium in the region sustaining the transmural patterns of reentry, as indicated in Fig. 5A. The AP traces in Fig. 5B show the development of large transmural V_m_ gradients (e.g. at 1831 ms) with fully depolarized epicardial cells (black traces) close to repolarizing endocardial tissue (green traces). The voltage gradients result in electrotonic current flowing towards the endocardial cells, and this facilitates the development of electrotonically-triggered EADs (Fig. 5B, green and blue traces from 1920 ms). This is a consequence of a weak repolarization reserve due to 50% I_Kr_ block and the electrotonic flow of current that allows for the increase in V_m_ during the plateau of the AP. The electrotonic current that facilitates EAD formation was estimated to be between 1 and 1.5 pA/pF in amplitude and 100–150 ms in duration by computing the diffusive part of the current in the monodomain equation.

**Fig. 6:**
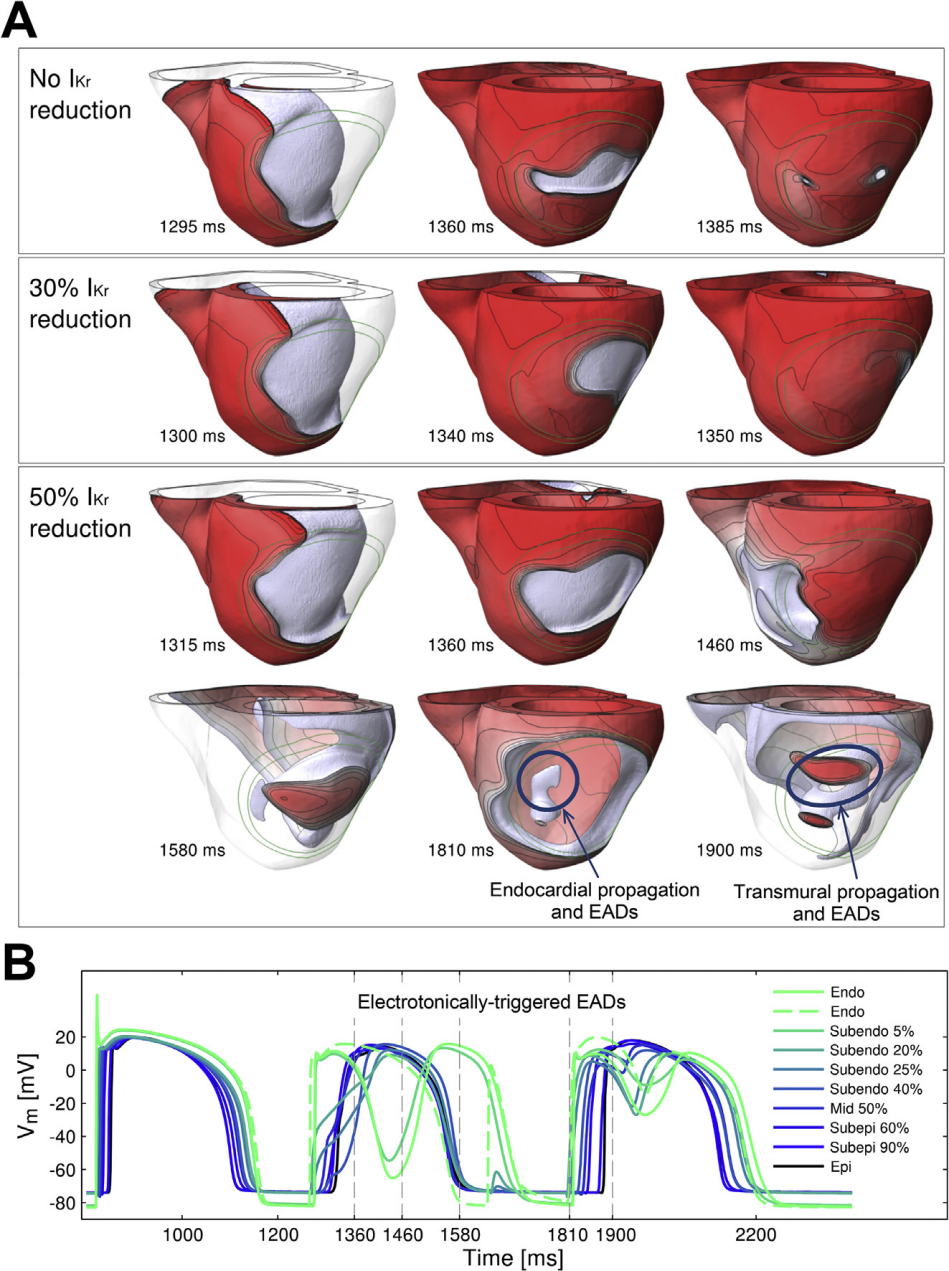
Intramural reentry is facilitated by prolonged APD due to EAD formation in the acutely-ischemic human ventricles with reduced repolarization reserve for long CI = 430 ms. A. Ectopic excitation at t = 1253 ms (corresponding to CI = 430 ms) fails to induce reentry for 0 and 30% I_Kr_ reduction (first and second row, respectively), but leads to establishment of transmural reentry for 50% I_Kr_ reduction (third and fourth row). B. Time course of the action potential in the area of transmural reentry for 50% I_Kr_ reduction marked with a blue circle in panel A. Legends indicate the transmural location for each trace (percentage of transmural distance from the endocardium). More details can be found in the supplemental figure.

In the absence of EAD formation triggered by electrotonic current, epicardial and endocardial cells would be refractory at the same time, and this would lead to conduction failure. However, as shown in Fig. 5B, EAD formation prolongs the time during which the endocardial cells are depolarized (green traces), increasing the time available to achieve the full recovery of neighbouring tissue (black and blue traces) and the continuation of the propagation, as was suggested by Kumar and Joyner, 1994, Pogwizd and Corr, 1990, Yan et al., 2001. This mechanism explains the increased propensity of reentry and VW extension for 50% compared to 30% I_Kr_ reduction, and is further illustrated in Fig. 6 through a comparison of simulations for strong I_Kr_ (0% reduction) and 30% and 50% reduction (top, middle and bottom panels). In all three cases, propagation first proceeds around the ICZ, and then through the ICZ once its tissue has recovered. For 0% and 30% I_Kr_ reduction, propagation completely engulfs the ventricles and reentry fails to be established. However, in the case of 50% I_Kr_ reduction, prolonged refractoriness in the ICZ leads to conduction block close to the BZ, followed by endocardial propagation and EAD formation (1810 ms and Fig. 6A and B), leading to transmural reentry as shown in Fig. 4, Fig. 5. More details can be found in the supplemental movies.

### 3.3 Prolonged low amplitude current may trigger EADs due to reactivation of the L-type calcium current gates

**Fig. 7:**
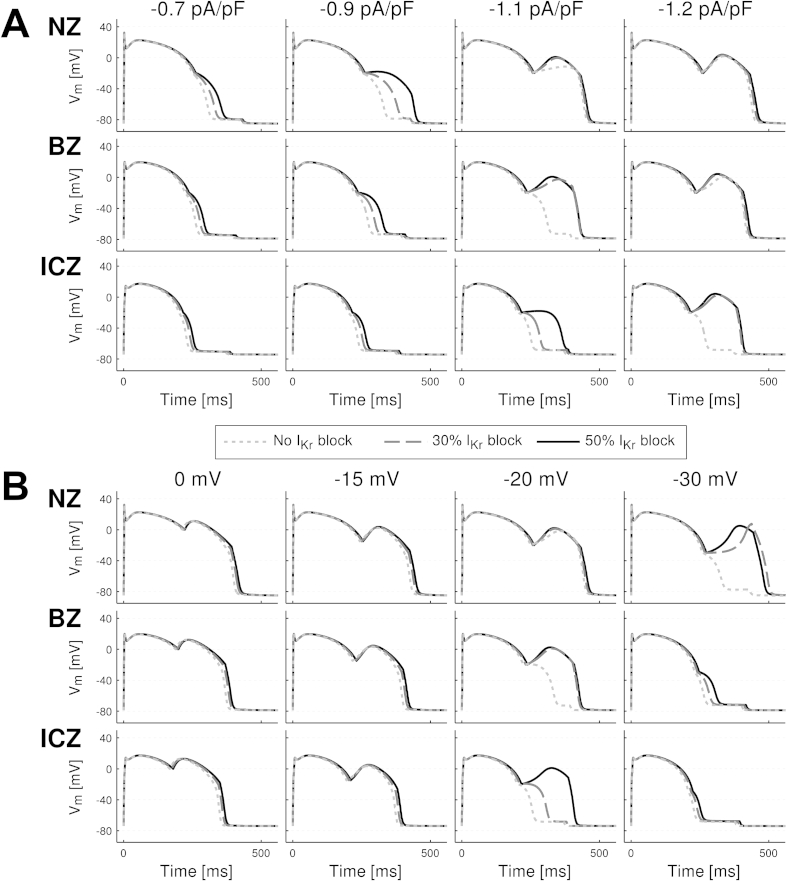
Characterization of EADs induced by low amplitude current injected during the repolarization phase for varying current amplitudes (A) and repolarization levels (B) for human ventricular cardiomyocytes representative of NZ (top), BZ (middle) and ICZ (bottom) for 0, 30 and 50% I_Kr_ reduction (short dash, long dash and continuous lines, respectively) (BZ: [K^+^]_o_ = 7 mmol/L, I_K(ATP)_ activation = 3%, I_Na_ and I_CaL_ peak conductance = 85% and ICZ: [K^+^]_o_ = 8.5 mmol/L, I_K(ATP)_ activation = 5%, I_Na_ and I_CaL_ peak conductance = 75%). In panel A, current is applied at −20 mV with amplitude 0.7, 0.9, 1.1 and 1.2 pA/pF (from left to right) and 170 ms duration. In panel B, current of 1.1 pA/pF amplitude and 170 ms duration is applied at different levels of repolarization from 0 to −40 mV transmembrane potential. The stimulation protocol consisted of a train of 100 stimuli with amplitude −30 pA/pF during 1 ms (1.3*threshold for AP trigger), at a CL of 500 ms.

**Fig. 8:**
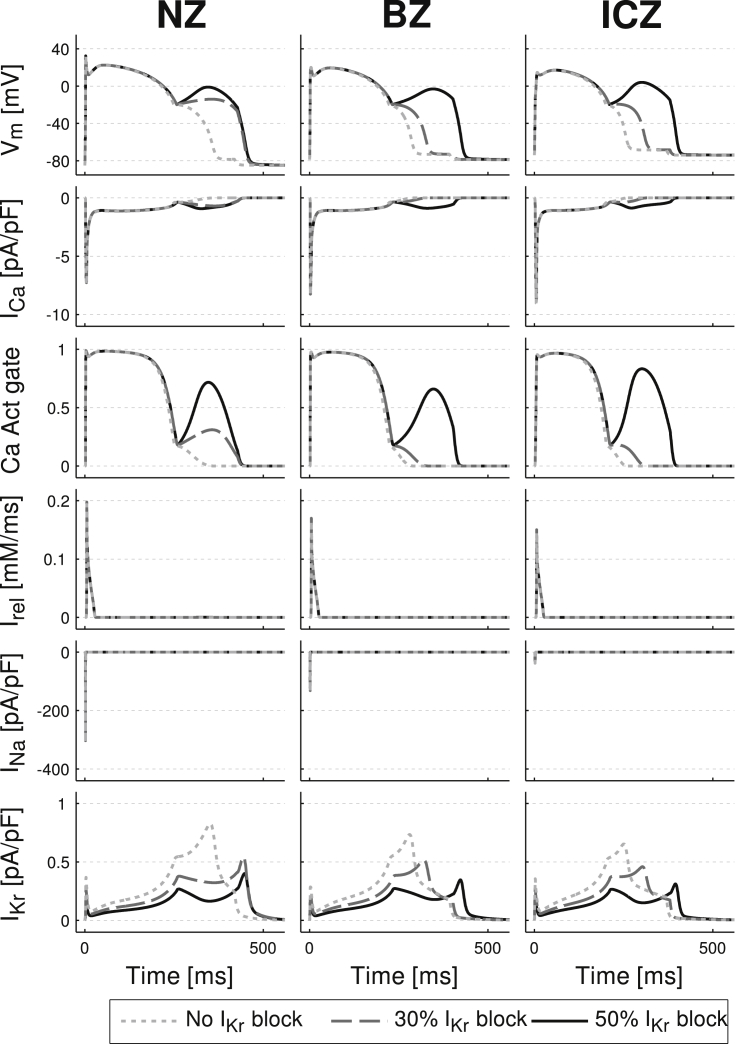
Ionic mechanisms underlying electrotonically-triggered EAD formation. From top to bottom: time course of the action potential, I_CaL_, I_CaL_ activation gate, calcium release current from the sarcoplasmic reticulum, I_Na_ and I_Kr_ for human ventricle cardiomyocytes representative of NZ, BZ and ICZ under varying degrees of repolarization reserve (0, 30 and 50% I_Kr_ reduction corresponding to short dash, long dash and continuous lines). Stimulation protocol and ischemic conditions as in Fig. 7.

In order to investigate the mechanisms underlying the formation of EADs in the acutely-ischemic human ventricles, we simulated the effect of a current injected during the repolarization phase on the human ventricular ionic dynamics and AP under normal and ischemic conditions with various degrees of repolarization reserve. Based on the conditions shown in Fig. 3, Fig. 4, Fig. 6, our hypothesis was that a current of low amplitude and prolonged duration could trigger L-type calcium current (I_CaL_) reactivation and EADs if injected during the repolarization phase. Results presented in Fig. 7, Fig. 8 show the following EAD mechanisms. Firstly, that EADs can be triggered when a current of low amplitude (less than 1/20th of the threshold for AP triggering) is injected in the NZ, BZ and ICZ human ventricle cardiomyocytes with normal and reduced repolarization reserve (Fig. 7). This is consistent with results in our ventricular simulations showing EAD formation across the BZ for 50% I_Kr_ block (Fig. 5, Fig. 6). Secondly, analysis of the ionic mechanisms underlying EAD formation shows that reactivation of the I_CaL_ current and its activation gate are the main driving force (Fig. 8, second and third row), while calcium release from the sarcoplasmic reticulum (as speculated by Verkerk et al. (2000)) is not involved (Fig. 8, fourth row). Thirdly, key to EAD formation is that the amplitude of the current injected is above a certain threshold. This is illustrated for example in the BZ and ICZ rows in Fig. 7A: a continuous 1.1 pA/pF current fails to develop EADs for normal and reduced repolarization reserve in the BZ and ICZ respectively, but they are triggered with an increased amplitude of 1.2 pA/pF. Furthermore, reduced repolarization reserve favors EAD occurrence, particularly when the prolonged current is applied during phase 3 of repolarization (i.e. for V_m_ ≤ −20 mV) (Fig. 6B). Indeed, the last two columns show that the occurrence of EADs is facilitated by I_Kr_ block. Low amplitude current applied at early stages of repolarization favors the occurrence of EAD formation compared to late repolarization stages (Fig. 8B). Fourthly, the window for EAD formation during the repolarization phase is less in the BZ and ICZ compared to NZ (Fig. 7B). Therefore, EADs occur for low amplitude current applied at membrane potentials from 0 to −35 mV in NZ cardiomyocytes, 0 to −25 mV in BZ cardiomyocytes and 0 to −20 mV in ICZ cardiomyocytes. Finally overall, ischemia reduces the propensity for EAD development (Fig. 7). This is due both to the stronger repolarization reserve in ischemia caused by I_K(ATP)_ activation and elevated extracellular potassium concentration ([K^+^]_o_). The latter decreases the excitability of the cell and therefore the likelihood of regenerative EAD-like behavior.

## 4 Discussion

High-resolution simulation data obtained with an anatomically-based model of the regionally-ischemic human ventricles reveal, for the first time, the electrotonic trigger of EADs and their contribution to transmural reentry and increased arrhythmic risk under conditions of reduced repolarization reserve. In our human ventricular model, EADs develop as a consequence of complex patterns of propagation, repolarization and current flow in the heterogeneous ischemic area, whereas they do not develop spontaneously in single cell or in homogeneous tissue with the same membrane kinetics. Simulations show that reduced I_Kr_ causes an anti-arrhythmic prolongation of refractoriness, which counteracts macro-reentry formation. However, it simultaneously causes a pro-arrhythmic reduction in repolarization reserve, which promotes EAD formation and transmural reentry. Arrhythmic risk in regional ischemia is therefore determined by a fine balance between macro-reentrant mechanisms driven by heterogeneity in refractoriness around the ischemic region, and micro-reentrant patterns, electrotonically-triggered EAD formation and transmural reentry. Reactivation of the L-type calcium current is the primary mechanism underlying EAD formation by prolonged low amplitude current injected during repolarization. Our investigations were primarily mechanistic, and thus conducted using a single human ventricles model given the computational demands. We identify key factors that will vary between individuals, and that may explain inter-subject differences in arrhythmic risk in acute ischemia modulated by repolarization reserve.

### 4.1 Computational model of the regionally-ischemic human ventricles and varied repolarization reserve

For this study, a novel human model was developed, which integrates the biophysical information on membrane kinetics and the pro-arrhythmic alterations induced by acute regional ischemia, as well as fiber orientation and realistic anatomy. Simulation results are in agreement with *in vivo* and *ex situ* human experimental studies including: human ventricular activation time (Durrer et al., 1970); inverse APD-activation time relationship (Hanson et al., 2009); ischemia-induced heterogeneity in AP, CV and ERP (Fig. 1, Fig. 2) (Carmeliet, 1999, Rodríguez et al., 2006); size of the ischemic region (Fig. 1) (Lee et al., 1981); pseudo-ECG, and its corresponding QT interval (414 ms), positive T wave, and T peak-to-Tend value (∼100 ms) (Fig. 1) (Browne et al., 1983). The agreement between experiments and simulations lends credibility to our new findings.

### 4.2 Macro and micro-reentry in the regionally-ischemic human ventricles

The macro-reentrant patterns of activation in the regionally-ischemic human ventricles model are also consistent with those reported in pig and canine hearts in the seminal work by Janse et al. (1980) and include three necessary conditions (Fig. 3). Firstly, the presence of conduction block is necessary and occurs when heterogeneity in refractoriness is such that at the time of ectopic excitation, NZ tissue is excitable and ICZ tissue refractory (ERP in ICZ > ERP in NZ). Secondly, retrograde conduction occurs when heterogeneity in refractoriness, ischemic area size and CV are such that propagation proceeds around the ICZ, while it remains blocked in the ICZ until ischemic tissue distal to the location of ectopic excitation recovers. A prolonged ERP in ischemic tissue would require increased ischemic region size or decreased CV to allow for retrograde conduction to occur. Finally, the reentry is complete once propagation has retrogradely traversed the ICZ and the tissue in the NZ has recovered. This condition depends on the ERP in the NZ, CV in the ICZ and the size of the ischemic region. A prolonged ERP in the NZ, as with I_Kr_ reduction, would be expected to decrease the likelihood of reentry.

Our simulation results show that I_Kr_ reduction slightly lowers the chances of meeting all three conditions for reentry by reducing the heterogeneity in ERP between the ICZ and NZ (from a range of 62–84 ms in control to 57–78 ms for 50% I_Kr_ reduction), while prolonging the ERP in both regions, therefore reducing the chances of retrograde propagation and reentry. This leads to a decrease in the VW for 30% I_Kr_ reduction but the trend becomes different with 50% I_Kr_ decrease. The main reason is the emergence of a newly identified pro-arrhythmic mechanism revealed by our simulations: transmural reentry sustained by electrotonically-triggered EAD development.

Even though Janse et al. also reported the occurrence of intramural reentry in some of their acute ischemia experiments in pig and dog, they were unable to resolve the underlying mechanisms due to the low resolution of their microelectrode recordings (Janse et al., 1980). Lukas and Antzelevitch also pointed at the possibility of phase 2 reentry in simulated ischemic tissue using thin epicardial sheets in acidotic “ischemic” solution and highlighted the need for validation of their hypothesis in “future studies using high-resolution techniques” (Lukas and Antzelevitch, 1996). Our simulations indeed yield high-resolution datasets consistent with a wide range of established knowledge, and reveal for the first time the development of electrotonically-triggered EADs implicated in intramural reentry caused by the heterogeneous disease substrate in the three-dimensional volume of the human ventricles.

### 4.3 Mechanisms of EAD development in tissue: electrotonic kick or intrinsic instability?

EADs have long been identified as a potentially important pro-arrhythmic player (Levy and Wiseman, 1991, Marban et al., 1986) and they have been demonstrated primarily in single cell studies with highly compromised repolarization reserve (caused by disease, mutations and/or pharmacological challenge) (Vandersickel et al., 2014, Zeng and Rudy, 1995). Despite tissue simulation studies showing their occurrence at the center of spiral wave rotors (Zemlin and Pertsov, 2007, Zemlin et al., 2009), their contribution to pro-arrhythmic tissue dynamics is still controversial, as electrotonic coupling has also been shown to suppress EAD formation and propagation (Huelsing et al., 2000, Mendez et al., 1969, Pueyo et al., 2011). Theoretical studies have demonstrated that EAD formation in tissue requires a huge reduction of repolarization reserve by simultaneously applying a 4-fold increase in I_CaL_ and 80% reduction of I_Kr_, as in Vandersickel et al. (2014). This may occur in certain extreme conditions, but it does not explain increased arrhythmic risk in disease conditions caused by mildly reduced repolarization reserve as in clinical trials in patients with coronary artery disease, but without genetic disorders (Steinberg et al., 2014).

Our simulations identify a more likely pro-arrhythmic mechanism of EAD formation and reentry, sustained by the heterogeneous ventricular substrate (Fig. 4, Fig. 5, Fig. 6). Reduced repolarization reserve facilitates EAD formation, but is not the primary trigger for repolarization instability, as spontaneous EADs do not occur in single cell or homogeneous tissue preparations with the same membrane kinetics. Our results show, for the first time, that EADs may be triggered in the ischemic human heart by the cell's environment in a heterogeneous substrate, which creates complex electrotonic flow of current across the BZ. Repolarization reserve and electrotonically-triggered EAD formation are therefore identified as potentially important determinants of pro-arrhythmic risk in the heterogeneous substrate of acute ischemia in human.

### 4.4 Implications

The mechanisms highlighted in our study reveal the complex balance of electrophysiological properties that determine pro-arrhythmic risk in acute regional ischemia. Macro-reentry is favored by both temporal and spatial aspects including ERP and CV, in both the NZ and ICZ, as well as the size of the ICZ and the occurrence of ectopic excitation. EAD formation leading to transmural reentry is determined by repolarization reserve and electrotonic current flowing from the heterogeneous substrate of the BZ. The anti-arrhythmic prolongation of ERP (by I_Kr_ reduction) indeed reduces the propensity for macro-reentry formation. However, the concomitant reduction of repolarization reserve is pro-arrhythmic through electrotonically-triggered EAD formation and establishment of transmural reentry. Our results therefore indicate that quantification of arrhythmic risk requires evaluation of both the heterogeneity of the tissue substrate and repolarization reserve, which are closely inter-related. Furthermore, evaluation of EAD mechanisms with pharmacological interventions requires consideration of the electrotonic trigger *via* injection of an external prolonged low amplitude current, as it would occur in tissue, rather than by only looking for spontaneous EAD formation.

Our study therefore provides a mechanistic explanation for the fact that interventions that reduce repolarization reserve and promote EAD formation are particularly dangerous in the presence of a heterogeneous substrate, as they may lead to re-entrant patterns. Other disease conditions such as hypertrophic cardiomyopathy and myocardial infarction could also provide a heterogeneous substrate (Roes et al., 2009, Schmidt et al., 2007). The key is to reduce heterogeneity as it provides the substrate for reentry and electrotonically-triggered EAD formation. This may explain the success of I_K(ATP)_ block in human hearts with cardiomyopathy, which was shown to reduce heterogeneity and promote spontaneous termination of ventricular fibrillation (Farid et al., 2011). Strategies for promoting sodium current activation could also be an anti-arrhythmic strategy (as was shown for myocardial infarction (Lau et al., 2009)), as they would increase wavelength and therefore reduce the chances of macro-reentry without affecting repolarization reserve. Conversely, pro-arrhythmic effects could be favored by interventions that reduce repolarization reserve without significant ERP prolongation, for example through multi-channel pharmacological action, or alternatively situations that further promote the heterogeneous substrate and reduce repolarization reserve, such as β-adrenergic stimulation. Increased calcium concentration may also facilitate the occurrence of EAD formation, for example during phase 1B of acute ischemia and following fast pacing (Huffaker et al., 2004).

The agreement with extensive experimental data supports the credibility of our regionally-ischemic human ventricles model and simulations, which also provide the flexibility required to evaluate the electrophysiological action of a range of pharmacological compounds and electrical therapies. The mechanistic understanding arising from such simulations could help in accelerating improvements in safety and efficacy of new anti-arrhythmic treatments. Furthermore, given the computational expense of the simulations in this study, we considered only one size, location and severity of the ischemic region and the membrane kinetics of the TP06 with 3 degrees of I_Kr_ block. This still enabled us to unravel key mechanisms determining pro-arrhythmic risk in ischemia and to demonstrate how mechanisms suggested in single cell or thin tissue layers could occur in the three-dimensional volume of the human ventricles. Our findings suggest that inter-subject differences in arrhythmic risk may be explained by differences not only in the ischemic region, but also in repolarization reserve, and further studies may be designed to examine patient-specific scenarios.

### 4.5 Limitations and additional potential players

Disagreements may arise with regards to the definition and root cause of EADs presented in this study. According to Weiss et al. “EADs occur in the setting of reduced repolarization reserve, [… ] such that the net outward current required to repolarize the myocyte is compromised. Under these conditions, any mechanism which regeneratively increases net inward current can potentially overcome and reverse repolarization. [… ] In the voltage range typical for EADs, the L-type Ca current (I_CaL_) and the Na–Ca exchange current (I_NCX_) are the major currents potentially meeting this positive feedback criterion” (Weiss et al., 2010). This definition is consistent with the EADs presented in our study: there is “reduced repolarization reserve” due to decreased I_Kr_ and the “net outward current required to repolarize the myocyte is compromised” due to I_CaL_ reactivation. Some studies, however, suggest that for EADs, I_CaL_ reactivation must occur solely due to intrinsic cell-scale mechanisms (January and Moscucci, 1992, Vandersickel et al., 2014), which in tissue may be suppressed due to intercellular coupling (Himel et al., 2013, Huelsing et al., 2000, Pueyo et al., 2011). Our study shows facilitation of EADs in tissue by electrotonic current, as has been suggested in coupled single cell experiments (Kumar and Joyner, 1994, Verkerk et al., 2000). Therefore, the definition of EADs presented here is consistent with known cell-scale mechanisms and is expanded to include the extrinsic drive provided by an electrotonic current.

Our study focuses on the quantification of the vulnerable window during which an ectopic beat can trigger a reentry, as the first condition for the establishment of ventricular tachycardia in regional ischemia. Our results however indicate that sustained ventricular tachycardia may require additional factors such as the occurrence of more than one ectopic beat and this could be the focus of further investigations as in Cao et al. (1999) and Nash et al. (2006). Furthermore, we evaluated the pro-arrhythmic substrate following ectopic excitation. The biophysical mechanisms leading to ectopic excitation, however, were not analyzed in our study. Previous studies support the role of Purkinje tissue as the source of spontaneous premature impulses (Janse et al., 1980). The Purkinje system was not included in our model, as it would greatly increase model complexity and assumptions with respect to the experimental data available. Future studies should extend our findings to evaluate the implication of the Purkinje system in providing the ectopic excitation and its potential role in contributing to arrhythmia complexity as pointed out in Behradfar et al. (2014). Furthermore, β-adrenergic stimulation is known to affect both repolarization reserve and heterogeneity through the modulation of key currents such as I_CaL_ and slowly activating delayed rectifier potassium current (I_Ks_). Further studies should investigate the potentially important implications of β-adrenergic stimulation in modulation of the pro-arrhythmic substrate characterized in our study. Additional sources of heterogeneity such as inter-ventricular differences in APD could also contribute to reentry in acute ischemia, especially if the ischemic region is located near the septum (Bueno-Orovio et al., 2012).

## Editors' note

Please see also related communications in this issue by Opthof et al. (2016) and Vigmond and Stuyvers (2016).

## Disclosure statement

None.

## Funding sources

This work was supported by a Wellcome Trust Fellowship in Basic Biomedical Sciences [grant number 100246/Z/12/Z to B.R.], an Engineering Physical Sciences Research Council doctoral scholarship to S.D. [EPSRC ref: EP/G03706X/1], the Canadian Institutes of Health Research [MOP 142424 to T.A.Q.] and the Nova Scotia Health Research Foundation [MED-EST-2014-9582 to T.A.Q], and a Marie Skłodowska-Curie Individual Fellowship from the H2020 EU Framework Programme for Research and Innovation [Proposal No: 655020-DTI4micro-MSCA-IF-EF-ST to E.Z.].

## Appendix A Supplementary data

**Supplemental Movie 1**. Distribution of V_m_ through time for 50% I_Kr_ block showing a normal heart beat followed by an ectopic beat (corresponding to CI = 361 ms). Bottom panel shows the corresponding pseudo-ECG computed for the electrode position shown in Fig. 1A. Snapshots of the movie are shown in Figs. 4 and 5 and a different view of the same simulation is shown in the following suppplemental movie.

**Supplemental Movie 2**. Distribution of depolarized tissue with V_m_ above −20 mV through time to emphasize intramural reentry patterns for 50% I_Kr_ block (with an ectopic beat applied at CI = 361 ms). Cells with V_m_.

Supplemental Figure. Complementary views of the snapshots shown in Fig. 6. Intramural reentry is facilitated by prolonged action potential duration (APD) due to electrotonically-triggered early afterdepolarization (EAD) formation in the acutely-ischemic human ventricles with reduced repolarization reserve (0, 30 and 50% block of the rapidly activating delayed rectifying potassium current (IKr)) for a long coupling interval (CI) of 430 ms. Ectopic excitation at t = 1253 ms (corresponding to CI = 430 ms) fails to induce reentry for 0 and 30% IKr reduction (first and second images, respectively), but leads to establishment of transmural reentry for 50% IKr reduction (last image). For each time point, three different views are shown, from left to right: the epicardium, the endocardium and the depolarized tissue with transmembrane voltage (Vm) above −20 mV (cells with Vm.

## References

[bib1] Behradfar E., Nygren A., Vigmond E.J. (2014). The role of Purkinje-myocardial coupling during ventricular arrhythmia: a modeling study. PLoS One.

[bib2] Bishop M., Gavaghan D., Trayanova N., Rodriguez B. (2007). Photon scattering effects in optical mapping of propagation and arrhythmogenesis in the heart. J. Electrocardiol..

[bib3] Browne K.F., Prystowsky E., Heger J.J., Chilson D.A., Zipes D.P. (1983). Prolongation of the Q-T interval in man during sleep. Am. J. Cardiol..

[bib4] Bueno-Orovio A., Hanson B.M., Gill J.S., Taggart P., Rodriguez B. (2012). In vivo human left-to-right ventricular differences in rate adaptation transiently increase pro-arrhythmic risk following rate acceleration. PloS One.

[bib5] Cao J.-M., Qu Z., Kim Y.-H., Wu T.-J., Garfinkel A., Weiss J.N., Karagueuzian H.S., Chen P.-S. (1999). Spatiotemporal heterogeneity in the induction of ventricular fibrillation by rapid pacing. Circ. Res..

[bib6] Carmeliet E. (1999). Cardiac ionic currents and acute ischemia: from channels to arrhythmias. Physiol. Rev..

[bib7] Coronel R., Fiolet J.W., Wilms-Schopman F.J., Schaapherder A.F., Johnson T.A., Gettes L.S., Janse M.J. (1988). Distribution of extracellular potassium and its relation to electrophysiologic changes during acute myocardial ischemia in the isolated perfused porcine heart. Circulation.

[bib8] Coronel R., Wilms-Schopman F.J., Opthof T., van Capelle F.J., Janse M.J. (1991). Injury current and gradients of diastolic stimulation threshold, TQ potential, and extracellular potassium concentration during acute regional ischemia in the isolated perfused pig heart. Circ. Res..

[bib9] Curtis M.J. (1998). Characterisation, utilisation and clinical relevance of isolated perfused heart models of ischaemia-induced ventricular fibrillation. Cardiovasc. Res..

[bib10] Durrer D.Th, Freud G.E., Janse M.J., Meijler F.L., Arzbaecher R.C. (1970). Total excitation of the isolated human heart. Circulation.

[bib11] Dutta S., Minchole A., Quinn T.A., Rodriguez B. (2013). Recent human ventricular cell action potential models under varied ischaemic conditions. Computing in Cardiology Conference (CinC), 2013.

[bib12] Edvardsson N., Hirsch I., Emanuelsson H., Pontén J., Olsson (1980). Sotalol-induced delayed ventricular repolarization in man. Eur. Heart J..

[bib13] Farid T., Nair K., Masse S., Azam M.A., Maguy A., Lai P.F.H., Umapathy K., Dorian P., Chauhan V., Varró A., Al-Hesayen A., Waxman M., Nattel S., Nanthakumar K. (2011). Role of KATP channels in the maintenance of ventricular fibrillation in cardiomyopathic human hearts. Circ. Res..

[bib14] Fossa A.A., Wisialowski T., Crimin K., Wolfgang E., Couderc J.-P., Hinterseer M., Kaab S., Zareba W., Badilini F., Sarapa N. (2007). Analyses of dynamic beat-to-beat QT–TQ interval (ECG Restitution) changes in humans under normal sinus rhythm and prior to an event of Torsades de Pointes during QT prolongation caused by Sotalol. Ann. Noninvasive Electrocardiol..

[bib15] Franz M.R., Bargheer K., Rafflenbeul W., Haverich A., Lichtlen P.R. (1987). Monophasic action potential mapping in human subjects with normal electrocardiograms: direct evidence for the genesis of the T wave. Circulation.

[bib16] Gima K., Rudy Y. (2002). Ionic current basis of electrocardiographic waveforms. Circ. Res..

[bib17] Glukhov A.V., Fedorov V.V., Lou Q., Ravikumar V.K., Kalish P.W., Schuessler R.B., Moazami N., Efimov I.R. (2010). Transmural dispersion of repolarization in failing and nonfailing human ventricle. Circ. Res..

[bib18] Hanson B., Sutton P., Elameri N., Gray M., Critchley H., Gill J.S., Taggart P. (2009). The interaction of activation-repolarisation coupling and restitution properties in humans. Circ. Arrhythm. Electrophysiol. CIRCEP.

[bib19] Himel H.D., Garny A., Noble P.J., Wadgaonkar R., Savarese J., Liu N., Bub G., El-Sherif N. (2013). Electrotonic suppression of early afterdepolarizations in the neonatal rat ventricular myocyte monolayer. J. Physiol..

[bib20] Huelsing D.J., Spitzer K.W., Pollard A.E. (2000). Electrotonic suppression of early afterdepolarizations in isolated rabbit Purkinje myocytes. Am. J. Physiol. Heart Circ. Physiol..

[bib21] Huffaker R., Lamp S.T., Weiss J.N., Kogan B. (2004). Intracellular calcium cycling, early afterdepolarizations, and reentry in simulated long QT syndrome. Heart rhythm...

[bib22] Janse M.J., Cinca J., Moréna H., Fiolet J.W., Kléber A.G., de Vries G.P., Becker A.E., Durrer D. (1979). The “border zone” in myocardial ischemia. An electrophysiological, metabolic, and histochemical correlation in the pig heart. Circ. Res..

[bib23] Janse M.J., van Capelle F.J., Morsink H., Kléber A.G., Wilms-Schopman F., Cardinal R., d' Alnoncourt C.N., Durrer D. (1980). Flow of “injury” current and patterns of excitation during early ventricular arrhythmias in acute regional myocardial ischemia in isolated porcine and canine hearts. Evidence for two different arrhythmogenic mechanisms. Circ. Res..

[bib24] Janse M.J., Wit A.L. (1989). Electrophysiological mechanisms of ventricular arrhythmias resulting from myocardial ischemia and infarction. Physiol. Rev..

[bib25] January C.T., Moscucci A. (1992). Cellular mechanisms of early afterdepolarizationsa. Ann. N. Y. Acad. Sci..

[bib26] Kazbanov I.V., Clayton R.H., Nash M.P., Bradley C.P., Paterson D.J., Hayward M.P., Taggart P., Panfilov A.V. (2014). Effect of global cardiac ischemia on human ventricular fibrillation: insights from a multi-scale mechanistic model of the human heart. PLoS Comput. Biol..

[bib27] Kumar R., Joyner R. (1994). An experimental model of the production of early after depolarizations by injury current from an ischemic region. Pflüg. Arch..

[bib28] Lau D.H., Clausen C., Sosunov E.A., Shlapakova I.N., Anyukhovsky E.P., Danilo P., Rosen T.S., Kelly C., Duffy H.S., Szabolcs M.J., Chen M., Robinson R.B., Lu J., Kumari S., Cohen I.S., Rosen M.R. (2009). Epicardial border zone overexpression of skeletal muscle sodium channel SkM1 normalizes activation, preserves conduction, and suppresses ventricular arrhythmia: an in silico, in vivo, in vitro study. Circulation.

[bib29] Lee J.T., Ideker R.E., Reimer K.A. (1981). Myocardial infarct size and location in relation to the coronary vascular bed at risk in man. Circulation.

[bib30] Levy M.N., Wiseman M.N. (1991). Electrophysiologic mechanisms for ventricular arrhythmias in left ventricular dysfunction: electrolytes, catecholamines and drugs. J. Clin. Pharmacol..

[bib31] Lukas A., Antzelevitch C. (1996). Phase 2 reentry as a mechanism of initiation of circus movement reentry in canine epicardium exposed to simulated ischemia. Cardiovasc. Res..

[bib32] Marban E., Robinson S.W., Wier W.G. (1986). Mechanisms of arrhythmogenic delayed and early afterdepolarizations in ferret ventricular muscle. J. Clin. Invest..

[bib33] Mendez C., Mueller W.J., Merideth J., Moe G.K. (1969). Interaction of transmembrane potentials in canine Purkinje fibers and at Purkinje fiber-muscle junctions. Circ. Res..

[bib34] Michailova A., Saucerman J., Belik M.E.E., McCulloch A.D. (2005). Modeling regulation of cardiac KATP and L-type Ca^2+^ currents by ATP, ADP, and Mg^2+^. Biophys. J..

[bib35] Nash M.P., Mourad A., Clayton R.H., Sutton P.M., Bradley C.P., Hayward M., Paterson D.J., Taggart P. (2006). Evidence for multiple mechanisms in human ventricular fibrillation. Circulation.

[bib57] Opthof T., Janse M.J., Meijborg V.M.F., Cinca J., Rosen M.R., Coronel R. (2016). Dispersion in ventricular repolarization in the human, canine and porcine heart. Prog. Biophys. Mol. Biol..

[bib36] Pogwizd S.M., Corr P.B. (1990). Mechanisms underlying the development of ventricular fibrillation during early myocardial ischemia. Circ. Res..

[bib37] Pueyo E., Corrias A., Virág L., Jost N., Szél T., Varró A., Szentandrássy N., Nánási P.P., Burrage K., Rodríguez B. (2011). A multiscale investigation of repolarization variability and its role in cardiac arrhythmogenesis. Biophys. J..

[bib38] Rodríguez B., Trayanova N., Noble D. (2006). Modeling cardiac ischemia. Ann. N. Y. Acad. Sci..

[bib39] Roes S.D., Borleffs J.W., van der Geest R.J., Westenberg J.J.M., Marsan N.A., Kaandorp T.A.M., Reiber J.H.C., Zeppenfeld K., Lamb H.J., de Roos A., Schalij M.J., Bax J.J. (2009). Infarct tissue heterogeneity assessed with contrast-enhanced MRI predicts spontaneous ventricular arrhythmia in patients with ischemic cardiomyopathy and implantable cardioverter-defibrillator. Circ. Cardiovasc. Imaging.

[bib40] Schmidt A., Azevedo C.F., Cheng A., Gupta S.N., Bluemke D.A., Foo T.K., Gerstenblith G., Weiss R.G., Marbán E., Tomaselli G.F., Lima J.A.C., Wu K.C. (2007). Infarct tissue heterogeneity by magnetic resonance imaging identifies enhanced cardiac arrhythmia susceptibility in patients with left ventricular dysfunction. Circulation.

[bib41] Spector P.S., Curran M.E., Keating M.T., Sanguinetti M.C. (1996). Class III antiarrhythmic drugs block HERG, a human cardiac delayed rectifier K+ channel. Open-channel block by methanesulfonanilides. Circ. Res..

[bib42] Steinberg B.A., Broderick S.H., Lopes R.D., Shaw L.K., Thomas K.L., DeWald T.A., Daubert J.P., Peterson E.D., Granger C.B., Piccini J.P. (2014). Use of antiarrhythmic drug therapy and clinical outcomes in older patients with concomitant atrial fibrillation and coronary artery disease. Europace.

[bib43] Streeter D.D., Spotnitz H.M., Patel D.P., Ross J., Sonnenblick E.H. (1969). Fiber orientation in the canine left ventricle during diastole and systole. Circ. Res..

[bib44] Sutton P.M., Taggart P., Opthof T., Coronel R., Trimlett R., Pugsley W., Kallis P. (2000). Repolarisation and refractoriness during early ischaemia in humans. Heart Br. Card. Soc..

[bib45] Taggart P., Sutton P.M.I., Opthof T., Coronel R., Trimlett R., Pugsley W., Kallis P. (2001). Transmural repolarisation in the left ventricle in humans during normoxia and ischaemia. Cardiovasc. Res..

[bib46] Tusscher K.H.W.J.T., Panfilov A.V. (2006). Alternans and spiral breakup in a human ventricular tissue model. Am. J. Physiol. Heart Circ. Physiol..

[bib47] Vandersickel N., Kazbanov I.V., Nuitermans A., Weise L.D., Pandit R., Panfilov A.V. (2014). A study of early afterdepolarizations in a model for human ventricular tissue. PLoS One.

[bib48] Verkerk A.O., Veldkamp M.W., de Jonge N., Wilders R., van Ginneken A.C.G. (2000). Injury current modulates afterdepolarizations in single human ventricular cells. Cardiovasc. Res..

[bib58] Vigmund E.J., Stuyvers B.D. (2016). Modelling or understanding of the His-Purkinje system. Prog. Biophys. Mol. Biol..

[bib49] Waldo A.L., Camm A.J., deRuyter H., Friedman P.L., MacNeil D.J., Pauls J.F., Pitt B., Pratt C.M., Schwartz P.J., Veltri E.P. (1996). Effect of d-sotalol on mortality in patients with left ventricular dysfunction after recent and remote myocardial infarction. The SWORD Investigators. Survival with Oral d-Sotalol. Lancet.

[bib50] Weiss J.N., Garfinkel A., Karagueuzian H.S., Chen P.-S., Qu Z. (2010). Early afterdepolarizations and cardiac arrhythmias. Heart Rhythm..

[bib51] Wilensky R.L., Tranum-Jensen J., Coronel R., Wilde A.A., Fiolet J.W., Janse M.J. (1986). The subendocardial border zone during acute ischemia of the rabbit heart: an electrophysiologic, metabolic, and morphologic correlative study. Circulation.

[bib52] Xie Y., Sato D., Garfinkel A., Qu Z., Weiss J.N. (2010). So little source, so much sink: requirements for afterdepolarizations to propagate in tissue. Biophys. J..

[bib53] Yan G.-X., Wu Y., Liu T., Wang J., Marinchak R.A., Kowey P.R. (2001). Phase 2 early afterdepolarization as a trigger of polymorphic ventricular tachycardia in acquired long-QT syndrome. Circulation.

[bib54] Zemlin C.W., Mitrea B.G., Pertsov A.M. (2009). Spontaneous onset of atrial fibrillation. Phys. Nonlinear Phenom..

[bib55] Zemlin C.W., Pertsov A.M. (2007). Bradycardic onset of spiral wave re-entry: structural substrates. Europace.

[bib56] Zeng J., Rudy Y. (1995). Early afterdepolarizations in cardiac myocytes: mechanism and rate dependence. Biophys. J..

