# Supplementary figures and images for: Early afterdepolarizations promote transmural reentry in ischemic human ventricles with reduced repolarization reserve
Source: Prog Biophys Mol Biol. 2016 Jan;120(1-3):236–48. doi: 10.1016/j.pbiomolbio.2016.01.008 (PMC4821233; doi:10.1016/j.pbiomolbio.2016.01.008)

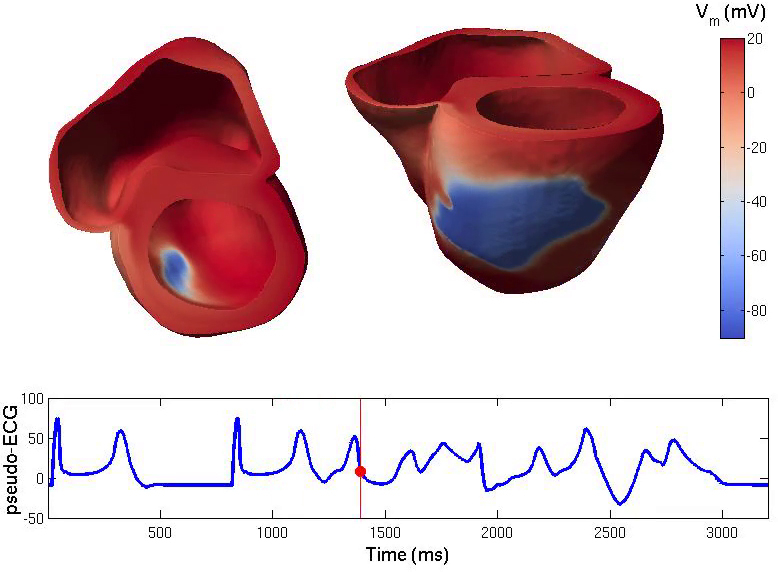

Supplement: Supplemental Movie 1. Distribution of Vm through time for 50% IKr block showing a normal heart beat followed by an ectopic beat (corresponding to CI = 361 ms). Bottom panel shows the corresponding pseudo-ECG computed for the electrode position shown in Fig. 1A. Snapshots of the movie are shown in Fi [file mmc1.jpg]

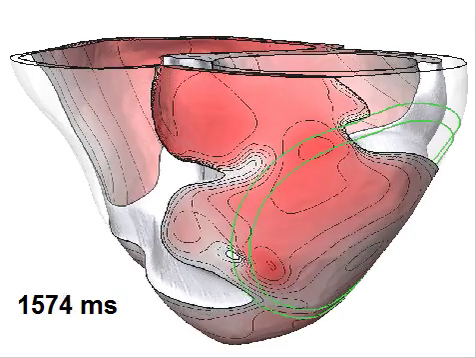

Supplement: Supplemental Movie 2. Distribution of depolarized tissue with Vm above −20 mV through time to emphasize intramural reentry patterns for 50% IKr block (with an ectopic beat applied at CI = 361 ms). Cells with Vm. [file mmc2.jpg]
