# Supplementary material for: Early afterdepolarizations promote transmural reentry in ischemic human ventricles with reduced repolarization reserve
Source: Prog Biophys Mol Biol. 2016 Jan;120(1-3):236–48. doi: 10.1016/j.pbiomolbio.2016.01.008 (PMC4821233; doi:10.1016/j.pbiomolbio.2016.01.008)
Supplement: Supplementary file 3 [file mmc3.docx]

**EXPANDED METHODS**

**1. Human ventricular model in regional acute myocardial ischemia**

An anatomically-based multiscale model of human regionally-ischemic ventricles was developed based on extensive experimental recordings from (Carmeliet, 1999; Coronel et al., 1991, 1988; Durrer et al., 1970; Edvardsson et al., 1980; Glukhov et al., 2010; Lee et al., 1981; Spector et al., 1996; Sutton et al., 2000; Taggart et al., 2001; Wilensky et al., 1986), including biophysically-detailed membrane kinetics, ischemia induced heterogeneities and fiber orientation (based on the Streeter method (Streeter et al., 1969)). Human ventricular membrane kinetics were simulated with the ten Tusscher 2006 action potential model (TP06) (Tusscher and Panfilov, 2006), extensively used in similar studies (Moreno et al., 2011; Wilhelms et al., 2012; Zemzemi et al., 2013) and shown to be suitable to simulate tachycardia and fibrillation as well as ischemic electrophysiological alterations in human (Dutta et al., 2013; Kazbanov et al., 2014). The ATP-sensitive potassium current (I_K(ATP)_) current was included in the TP06 model, using the description in (Michailova et al., 2005).

**2. Heterogeneous electrophysiological substrate in the ischemic region**

Size and location of ischemic regions exhibit great variability. We represented the ischemic region in our model as affecting ~40% of the left ventricle, which is within the range of 13-72% reported by Lee et al. (Lee et al., 1981) (Figure 1A). Furthermore studies in animal have shown that the likelihood of ventricular fibrillation is the greatest when the ischemic region extends about 40-50% of the ventricles, as reviewed in (Curtis, 1998). The ischemic region was modeled in agreement with experimental findings by (Coronel et al., 1991; Heidenreich et al., 2012; Rodríguez et al., 2006; Wilensky et al., 1986) including: (i) the ischemic central zone of radius 3 cm (ICZ); (ii) the border zone (BZ), representing a heterogeneous 0.5 cm wide band of tissue surrounding the ICZ, caused by diffusion between ICZ and normal tissue, (iii) and the endocardial BZ, a layer of healthy tissue of width 0.1 cm in the endocardium caused by diffusion of oxygen and nutrients from the blood in the ventricular cavity (Janse et al., 1986; Wilensky et al., 1986).

In the ICZ, the ionic changes caused by the three main components of acute ischemia (namely hyperkalemia, hypoxia and acidosis) were introduced as in (Coronel et al., 1991; Heidenreich et al., 2012; Rodríguez et al., 2006; Wilensky et al., 1986). We applied a degree of ischemic severity that corresponds to highest arrhythmic risk (Carmeliet, 1999; Janse and Wit, 1989; Kazbanov et al., 2014; Rodríguez et al., 2006), and which coincides with early stages of ischemia (up to 15 min). Therefore, ischemic cells suffer from increased extracellular potassium concentration ([K^+^]_o_) of 8.5 mmol/L (Clayton et al., 2011; Heidenreich et al., 2012; Vermeulen, 1996; Wilde et al., 1990), increased I_K(ATP)_ by 5% caused by hypoxia, and decreased peak conductance of fast sodium current (I_Na_) and L-type calcium current (I_CaL_) by 25% due to acidosis (Coronel et al., 1988; Rodríguez et al., 2006; Wilensky et al., 1986). The BZ included a linear gradient in electrophysiological properties from the ICZ to the normal zone (NZ) tissue as shown in experiments (Coronel et al., 1988; Wilensky et al., 1986). We did not include calcium overload or gap junction changes as they occur at later stages of ischemia (Carmeliet, 1999), they could be considered in later studies. NZ tissue had a [K^+^]_o_ of 5.4 mmol/L (original value given in the TP06 model), zero I_K(ATP)_ and the default conductances for I_Na_ and I_CaL_ in the TP06 model.

The human model also included transmural heterogeneities in action potential duration (APD), measured in experimental and clinical studies ranging from 10-20 ms *in vivo* (Taggart et al., 2001) to 100 ms *in vitro* in human (Glukhov et al., 2010; Kuo et al., 1983). Therefore, transmural differences in the slowly activating delayed rectifier potassium current (I_Ks_) were considered as in (Péréon et al., 2000; Xu et al., 2001), by applying a 6:4 endocardium to epicardium ratio of I_Ks_ maximal conductance (Tusscher and Panfilov, 2006). These changes were applied uniformly throughout the ventricles, including in the ischemic region. As the existence of longer subendocardial APD remains controversial (Glukhov et al., 2010; Péréon et al., 2000; Taggart et al., 2001, p. 200; Yan et al., 1998), a linear transmural APD gradient from endocardium to epicardium was imposed, with the longest APD at the endocardium (Glukhov et al., 2010; Taggart et al., 2001). The APD gradient was 17% of the maximum APD, which is between values of 10% obtained by Taggart et al. from *in vivo* human studies (Taggart et al., 2001) and 24% obtained by Glukhov et al. from *in vitro* human wedge preparations (Glukhov et al., 2010). The model reproduced the physiological inverse APD - activation time relationship resulting in a positive T wave in the pseudo-ECG, as shown in data obtained in *in vivo* human hearts (Franz et al., 1987) and also in intact dog hearts (Spach and Barr, 1975).

**3. Stimulation protocol**

Purkinje-like activation was simulated by stimulating the endocardium to mimic the activation sequence in (Durrer et al., 1970), and total ventricular activation time was 63 ms. After 20 ms at rest, endocardial stimulation (S1) was applied twice with a cycle length (CL) of 800 ms. Then, a premature excitation (S2) of 2 cm was applied in a region close to the BZ (Figure 1A), which included the NZ and the ICZ, prone to premature excitation and mimicking findings by Janse et al. (Bernus et al., 2005; Coronel et al., 2002). The coupling interval (CI) of the premature stimulus (e.g. time interval difference between S1 and S2) was varied to quantify the vulnerability window (VW) of reentry, computed as the range of CIs that resulted in reentry (calculated with 1 ms precision and defined as one or more completed cycles of propagation around the ischemic region).

As illustrated in Figure 1, the human ventricular model reproduced the reported electrophysiological heterogeneity caused by regional ischemia, including elevated resting transmembrane potential (V_m_) and short APD (Bernus et al., 2005; Heidenreich et al., 2012; Rodríguez et al., 2006) in the ICZ with respect to NZ, in agreement with *in vivo* human and *ex situ* animal studies (Carmeliet, 1999; Sutton et al., 2000, p. 200; Taggart et al., 2001; Wilensky et al., 1986). Resting V_m_ in the ICZ was -70 mV compared to -86 mV in the NZ, and APD was 30% shorter in the ICZ than in the NZ, close to the 35% difference reported in human *in vivo* measurements (Sutton et al., 2000). The simulated pseudo-ECG was computed as in (Gima and Rudy, 2002), as the extracellular unipolar potential at a position 3.6 cm away from the epicardial surface (Figure 1A), and yielded a physiological QT interval of ~400 ms and a positive T wave, as described in *in vivo* human hearts and computationally (Franz et al., 1987; Gima and Rudy, 2002).

**4. Reduced repolarization reserve**

In order to investigate implications of reduced repolarization reserve in acute ischemia, we considered three levels of repolarization reserve, represented by 0, 30 and 50% decrease in the TP06 rapidly activating delayed rectifying potassium current (I_Kr_) conductance. These changes were applied uniformly throughout the ventricles, including in the ischemic region. The levels of I_Kr_ block were chosen to induce changes in APD similar to the minimum and maximum of effective free therapeutic plasma concentrations (EFTPC) of sotalol, described by Redfern et al. as approximately 2 μM and 15 μM respectively (this is equivalent to plasma concentrations of approximately 0.5 μg/mL and 4.1 μg/mL given a sotalol molecular weight of 272.36 g/mol) (Nademanee et al., 1985; Redfern et al., 2003; Ward et al., 1979). The simulations yield a 5% and 9% prolongation in QT in the pseudo-ECG, which is similar to the 7% and 12% increase found by Fossa *et al.* in healthy patients, 2-4 h after being given 160 mg and 320 mg of sotalol respectively (Fossa et al., 2007).

One-dimensional simulations were conducted to characterize the effect of reduced repolarization reserve in NZ and ICZ tissue for varying degrees of I_Kr_ reduction. APD was calculated at 90% repolarization at steady state for each CL. Effective refractory period (ERP) was evaluated after reaching steady state at each CL and calculated as the shortest CI to induce sustained propagation in the tissue. Conduction velocity (CV) was calculated at steady state from the time interval separating two activation times at two points in the center of the tissue separated by 2 mm.

As shown in Figure 2, simulations yield the known electrophysiological effects of acute ischemia, including APD shortening, ERP prolongation and slow CV for all degrees of repolarization reserve. Note that no EADs occurred in any of the single cell and one-dimensional simulations.

**5. Numerical Methods**

Simulations were run in Chaste (Mirams et al., 2013; Pitt-Francis et al., 2009) on the HECToR UK National supercomputer. At the cell level, the biophysical activity of the ionic currents and concentrations across the cell membrane were modeled by a set of ordinary differential equations (ODEs) defined in the TP06 model. The backward Euler method was used to solve these ODEs. At the tissue level, the monodomain model described the electrical activity of the myocardium, through a parabolic partial differential equation (PDE). To solve the monodomain model we first established a discretization in time, using semi-implicit time discretization, and in space, using the finite element method (FEM). The latter method divides the continuous domain into discrete sub-domains called elements, in this case tetrahedra. Inside each element, the solution was approximated by an interpolation function. These simulation techniques have been extensively described in the following studies (Bernabeu et al., 2014; Niederer et al., 2011; Pathmanathan et al., 2010). Using 1024 processors on the HECToR UK National supercomputer, simulations of 800 ms of ventricular activity took approximately 6 h of supercomputing time.

The image-based human anatomical mesh contains 2.5 M nodes with 400 μm spatial discretization, required for convergence of the numerical algorithms (Bernabeu et al., 2010). As shown in the benchmarking study by Niederer et al., Chaste shows the same convergence with a mesh discretization of 500 μM as with 100 μM (Niederer et al., 2011). Furthermore, our simulation study in a 5 cm long fiber of cells under ischemic conditions and reduced repolarization reserve showed a change of 6% or less in CV when mesh discretization was varied from 100 μM to 400 μM.**References**

Bernabeu, M.O., Southern, J., Wilson, N., Strazdins, P., Cooper, J., Pitt-Francis, J., 2014. Chaste. Int. J. High Perform. Comput. Appl. 28, 13–32. doi:10.1177/1094342012474997

Bernabeu, M.O., Wallman, M., Rodriguez, B., 2010. Shock-induced arrhythmogenesis in the human heart: A computational modelling study. Conf. Proc. Annu. Int. Conf. IEEE Eng. Med. Biol. Soc. IEEE Eng. Med. Biol. Soc. Conf. 2010, 760–763. doi:10.1109/iembs.2010.5626338

Bernus, O., Zemlin, C.W., Zaritsky, R.M., Mironov, S.F., Pertsov, A.M., 2005. Alternating conduction in the ischaemic border zone as precursor of reentrant arrhythmias: A simulation study. Europace 7, S93–S104. doi:10.1016/j.eupc.2005.03.018

Carmeliet, E., 1999. Cardiac ionic currents and acute ischemia: from channels to arrhythmias. Physiol. Rev. 79, 917–1017.

Clayton, R.H., Nash, M.P., Bradley, C.P., Panfilov, A.V., Paterson, D.J., Taggart, P., 2011. Experiment-model interaction for analysis of epicardial activation during human ventricular fibrillation with global myocardial ischaemia. Prog. Biophys. Mol. Biol. doi:10.1016/j.pbiomolbio.2011.06.010

Coronel, R., Fiolet, J.W., Wilms-Schopman, F.J., Schaapherder, A.F., Johnson, T.A., Gettes, L.S., Janse, M.J., 1988. Distribution of extracellular potassium and its relation to electrophysiologic changes during acute myocardial ischemia in the isolated perfused porcine heart. Circulation 77, 1125–1138.

Coronel, R., Wilms-Schopman, F.J., Opthof, T., van Capelle, F.J., Janse, M.J., 1991. Injury current and gradients of diastolic stimulation threshold, TQ potential, and extracellular potassium concentration during acute regional ischemia in the isolated perfused pig heart. Circ Res 68, 1241–1249.

Coronel, R., Wilms-Schopman, F.J.G., deGroot, J.R., 2002. Origin of ischemia-induced phase 1b ventricular arrhythmias in pig hearts. J Am Coll Cardiol 39, 166–176.

Curtis, M.J., 1998. Characterisation, utilisation and clinical relevance of isolated perfused heart models of ischaemia-induced ventricular fibrillation. Cardiovasc. Res. 39, 194–215. doi:10.1016/s0008-6363(98)00083-2

Durrer, D., Th, Freud, G.E., Janse, M.J., Meijler, F.L., Arzbaecher, R.C., 1970. Total Excitation of the Isolated Human Heart. Circulation 41, 899–912. doi:10.1161/01.cir.41.6.899

Dutta, S., Minchole, A., Quinn, T.A., Rodriguez, B., 2013. Recent human ventricular cell action potential models under varied ischaemic conditions, in: Computing in Cardiology Conference (CinC), 2013. IEEE, pp. 695–698.

Edvardsson, N., Hirsch, I., Emanuelsson, H., Pontén, J., Olsson, 1980. Sotalol-induced delayed ventricular repolarization in man. Eur. Heart J. 1, 335–343. doi:10.1093/eurheartj/1.5.335

Fossa, A.A., Wisialowski, T., Crimin, K., Wolfgang, E., Couderc, J.-P., Hinterseer, M., Kaab, S., Zareba, W., Badilini, F., Sarapa, N., 2007. Analyses of Dynamic Beat-to-Beat QT–TQ Interval (ECG Restitution) Changes in Humans under Normal Sinus Rhythm and Prior to an Event of Torsades de Pointes during QT Prolongation Caused by Sotalol. Ann. Noninvasive Electrocardiol. 12, 338–348. doi:10.1111/j.1542-474x.2007.00183.x

Franz, M.R., Bargheer, K., Rafflenbeul, W., Haverich, A., Lichtlen, P.R., 1987. Monophasic action potential mapping in human subjects with normal electrocardiograms: direct evidence for the genesis of the T wave. Circulation 75, 379–386. doi:10.1161/01.cir.75.2.379

Gima, K., Rudy, Y., 2002. Ionic Current Basis of Electrocardiographic Waveforms. Circ. Res. 90, 889–896. doi:10.1161/01.res.0000016960.61087.86

Glukhov, A.V., Fedorov, V.V., Lou, Q., Ravikumar, V.K., Kalish, P.W., Schuessler, R.B., Moazami, N., Efimov, I.R., 2010. Transmural Dispersion of Repolarization in Failing and Nonfailing Human Ventricle. Circ. Res. 106, 981–991. doi:10.1161/circresaha.109.204891

Heidenreich, E.A., Ferrero, J.M., Rodríguez, J.F., 2012. Modeling the Human Heart Under Acute Ischemia - Springer, in: Springer (Ed.), .

Janse, M., Kleber, A., Capucci, A., Coronel, R., Wilmsschopman, F., 1986. Electrophysiological basis for arrhythmias caused by acute ischemiaRole of the subendocardium. J. Mol. Cell. Cardiol. 18, 339–355. doi:10.1016/s0022-2828(86)80898-7

Janse, M.J., Wit, A.L., 1989. Electrophysiological mechanisms of ventricular arrhythmias resulting from myocardial ischemia and infarction. Physiol. Rev. 69, 1049–1169.

Kazbanov, I.V., Clayton, R.H., Nash, M.P., Bradley, C.P., Paterson, D.J., Hayward, M.P., Taggart, P., Panfilov, A.V., 2014. Effect of global cardiac ischemia on human ventricular fibrillation: insights from a multi-scale mechanistic model of the human heart. PLoS Comput. Biol. 10.

Kuo, C.S., Munakata, K., Reddy, C.P., Surawicz, B., 1983. Characteristics and possible mechanism of ventricular arrhythmia dependent on the dispersion of action potential durations. Circulation 67, 1356–1367.

Lee, J.T., Ideker, R.E., Reimer, K.A., 1981. Myocardial infarct size and location in relation to the coronary vascular bed at risk in man. Circulation 64, 526–534. doi:10.1161/01.cir.64.3.526

Michailova, A., Saucerman, J., Belik, M.E.E., McCulloch, A.D., 2005. Modeling regulation of cardiac KATP and L-type Ca2+ currents by ATP, ADP, and Mg2+. Biophys. J. 88, 2234–2249. doi:10.1529/biophysj.104.046284

Mirams, G.R., Arthurs, C.J., Bernabeu, M.O., Bordas, R., Cooper, J., Corrias, A., Davit, Y., Dunn, S.-J., Fletcher, A.G., Harvey, D.G., Marsh, M.E., Osborne, J.M., Pathmanathan, P., Pitt-Francis, J., Southern, J., Zemzemi, N., Gavaghan, D.J., 2013. Chaste: An Open Source C++ Library for Computational Physiology and Biology. PLoS Comput Biol 9, e1002970+. doi:10.1371/journal.pcbi.1002970

Moreno, J.D., Zhu, Z.I., Yang, P.-C., Bankston, J.R., Jeng, M.-T., Kang, C., Wang, L., Bayer, J.D., Christini, D.J., Trayanova, N.A., Ripplinger, C.M., Kass, R.S., Clancy, C.E., 2011. A Computational Model to Predict the Effects of Class I Anti-Arrhythmic Drugs on Ventricular Rhythms. Sci. Transl. Med. 3, 98ra83. doi:10.1126/scitranslmed.3002588

Nademanee, K., Feld, G., Hendrickson, J., Singh, P.N., Singh, B.N., 1985. Electrophysiologic and antiarrhythmic effects of sotalol in patients with life-threatening ventricular tachyarrhythmias. Circulation 72, 555–564. doi:10.1161/01.cir.72.3.555

Niederer, S.A., Kerfoot, E., Benson, A.P., Bernabeu, M.O., Bernus, O., Bradley, C., Cherry, E.M., Clayton, R., Fenton, F.H., Garny, A., Heidenreich, E., Land, S., Maleckar, M., Pathmanathan, P., Plank, G., Rodríguez, J.F., Roy, I., Sachse, F.B., Seemann, G., Skavhaug, O., Smith, N.P., 2011. Verification of cardiac tissue electrophysiology simulators using an N-version benchmark. Philos. Trans. R. Soc. Math. Phys. Eng. Sci. 369, 4331–4351. doi:10.1098/rsta.2011.0139

Pathmanathan, P., Bernabeu, M.O., Bordas, R., Cooper, J., Garny, A., Pitt-Francis, J.M., Whiteley, J.P., Gavaghan, D.J., 2010. A numerical guide to the solution of the bi-domain equations of cardiac electrophysiology. Prog. Biophys. Mol. Biol. 102, 136–155. doi:10.1016/j.pbiomolbio.2010.05.006

Péréon, Y., Demolombe, S., Baró, I., Drouin, E., Charpentier, F., Escande, D., 2000. Differential expression of KvLQT1 isoforms across the human ventricular wall. Am. J. Physiol. Heart Circ. Physiol. 278.

Pitt-Francis, J., Pathmanathan, P., Bernabeu, M.O., Bordas, R., Cooper, J., Fletcher, A.G., Mirams, G.R., Murray, P., Osborne, J.M., Walter, A., Chapman, S.J., Garny, A., van Leeuwen, I.M.M., Maini, P.K., Rodríguez, B., Waters, S.L., Whiteley, J.P., Byrne, H.M., Gavaghan, D.J., 2009. Chaste: A test-driven approach to software development for biological modelling. Comput. Phys. Commun. 180, 2452–2471. doi:10.1016/j.cpc.2009.07.019

Redfern, W.S., Carlsson, L., Davis, A.S., Lynch, W.G., MacKenzie, I., Palethorpe, S., Siegl, P.K.S., Strang, I., Sullivan, A.T., Wallis, R., Camm, A.J., Hammond, T.G., 2003. Relationships between preclinical cardiac electrophysiology, clinical QT interval prolongation and torsade de pointes for a broad range of drugs: evidence for a provisional safety margin in drug development. Cardiovasc. Res. 58, 32–45. doi:10.1016/s0008-6363(02)00846-5

Rodríguez, B., Trayanova, N., Noble, D., 2006. Modeling Cardiac Ischemia. Ann. N. Y. Acad. Sci. 1080, 395–414. doi:10.1196/annals.1380.029

Spach, M.S., Barr, R.C., 1975. Ventricular intramural and epicardial potential distributions during ventricular activation and repolarization in the intact dog. Circ Res 37, 243–257. doi:10.1161/01.res.37.2.243

Spector, P.S., Curran, M.E., Keating, M.T., Sanguinetti, M.C., 1996. Class III antiarrhythmic drugs block HERG, a human cardiac delayed rectifier K+ channel. Open-channel block by methanesulfonanilides. Circ. Res. 78, 499–503. doi:10.1161/01.res.78.3.499

Streeter, D.D., Spotnitz, H.M., Patel, D.P., Ross, J., Sonnenblick, E.H., 1969. Fiber Orientation in the Canine Left Ventricle during Diastole and Systole. Circ. Res. 24, 339–347.

Sutton, P.M., Taggart, P., Opthof, T., Coronel, R., Trimlett, R., Pugsley, W., Kallis, P., 2000. Repolarisation and refractoriness during early ischaemia in humans. Heart Br. Card. Soc. 84, 365–369.

Taggart, P., Sutton, P.M.I., Opthof, T., Coronel, R., Trimlett, R., Pugsley, W., Kallis, P., 2001. Transmural repolarisation in the left ventricle in humans during normoxia and ischaemia. Cardiovasc. Res. 50, 454–462. doi:10.1016/s0008-6363(01)00223-1

Tusscher, K.H.W.J.T., Panfilov, A.V., 2006. Alternans and spiral breakup in a human ventricular tissue model. Am. J. Physiol. - Heart Circ. Physiol. 291, H1088–H1100. doi:10.1152/ajpheart.00109.2006

Vermeulen, J., 1996. Electrophysiologic and Extracellular Ionic Changes During Acute Ischemia in Failing and Normal Rabbit Myocardium. J. Mol. Cell. Cardiol. 28, 123–131. doi:10.1006/jmcc.1996.0012

Ward, D.E., Camm, A.J., Spurrell, R.A.J., 1979. The acute cardiac electrophysiological effects of intravenous sotalol hydrochloride. Clin Cardiol 2, 185–191. doi:10.1002/clc.4960020303

Wilde, A.A., Escande, D., Schumacher, C.A., Thuringer, D., Mestre, M., Fiolet, J.W., Janse, M.J., 1990. Potassium accumulation in the globally ischemic mammalian heart. A role for the ATP-sensitive potassium channel. Circ. Res. 67, 835–843.

Wilensky, R.L., Tranum-Jensen, J., Coronel, R., Wilde, A.A., Fiolet, J.W., Janse, M.J., 1986. The subendocardial border zone during acute ischemia of the rabbit heart: an electrophysiologic, metabolic, and morphologic correlative study. Circulation 74, 1137–1146.

Wilhelms, M., Rombach, C., Scholz, E.P., Dössel, O., Seemann, G., 2012. Impact of amiodarone and cisapride on simulated human ventricular electrophysiology and electrocardiograms. Europace 14, v90–v96. doi:10.1093/europace/eus281

Xu, X., Rials, S.J., Wu, Y., Salata, J.J., Liu, T., Bharucha, D.B., Marinchak, R.A., Kowey, P.R., 2001. Left Ventricular Hypertrophy Decreases Slowly but Not Rapidly Activating Delayed Rectifier Potassium Currents of Epicardial and Endocardial Myocytes in Rabbits. Circulation 103, 1585–1590. doi:10.1161/01.cir.103.11.1585

Yan, G.-X., Shimizu, W., Antzelevitch, C., 1998. Characteristics and Distribution of M Cells in Arterially Perfused Canine Left Ventricular Wedge Preparations. Circulation 98, 1921–1927. doi:10.1161/01.cir.98.18.1921

Zemzemi, N., Bernabeu, M.O., Saiz, J., Cooper, J., Pathmanathan, P., Mirams, G.R., Pitt-Francis, J., Rodriguez, B., 2013. Computational assessment of drug-induced effects on the electrocardiogram: from ion channel to body surface potentials. Br J Pharmacol 168, 718–733. doi:10.1111/j.1476-5381.2012.02200.x
