# Supplementary material for: Early afterdepolarizations promote transmural reentry in ischemic human ventricles with reduced repolarization reserve
Source: Prog Biophys Mol Biol. 2016 Jan;120(1-3):236–48. doi: 10.1016/j.pbiomolbio.2016.01.008 (PMC4821233; doi:10.1016/j.pbiomolbio.2016.01.008)
Supplement: Supplemental Figure. Complementary views of the snapshots shown in Fig. 6. Intramural reentry is facilitated by prolonged action potential duration (APD) due to electrotonically-triggered early afterdepolarization (EAD) formation in the acutely-ischemic human ventricles with reduced repolarization r [file mmc4.pdf]

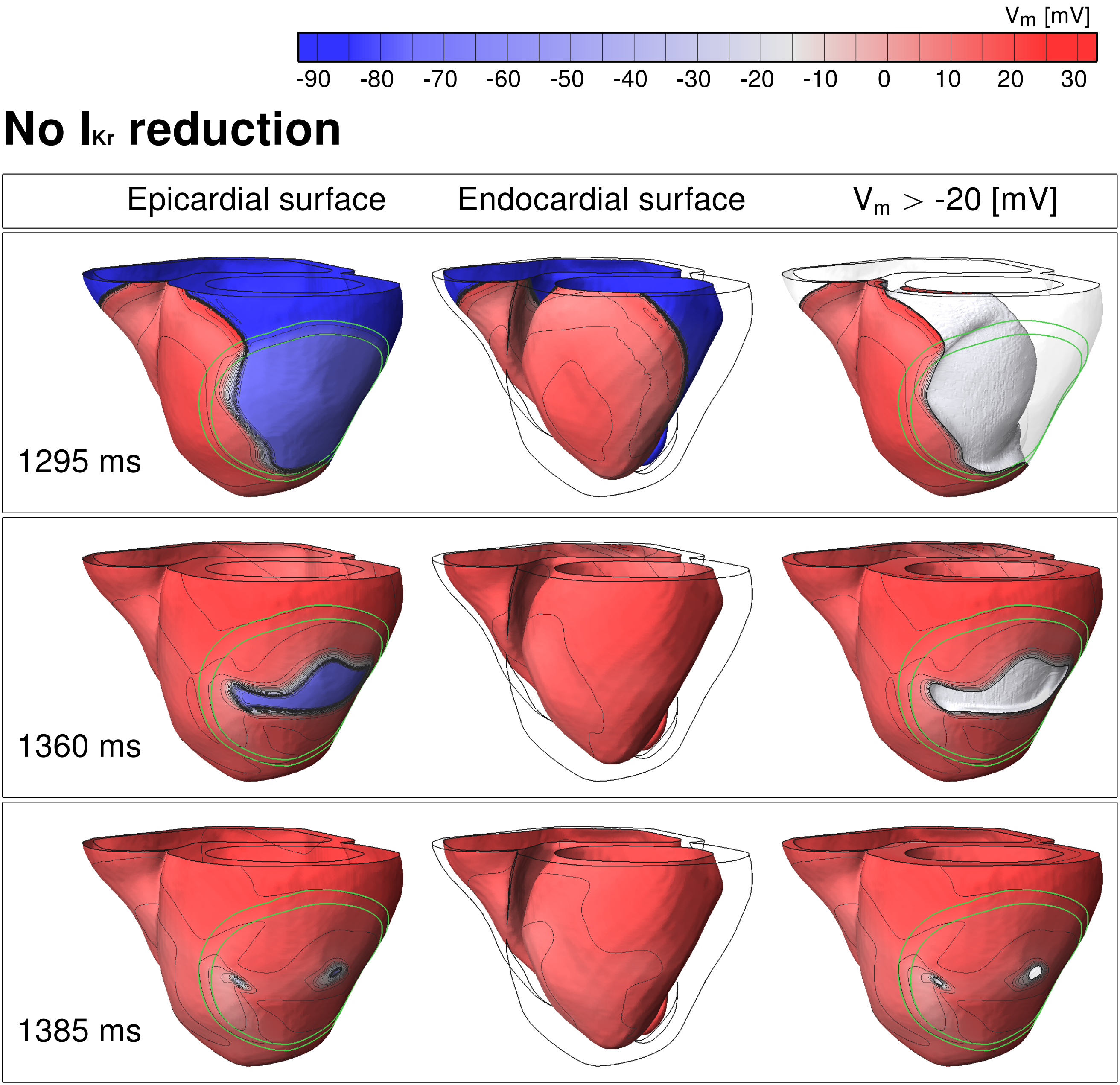

Vm [mV]
-90
-80
-70
-60
-50
-40
-30
-20
-10
0
10
20
30
No IKr reduction
Epicardial surface
Endocardial surface
Vm > -20 [mV]
1295 ms
1360 ms
1385 ms

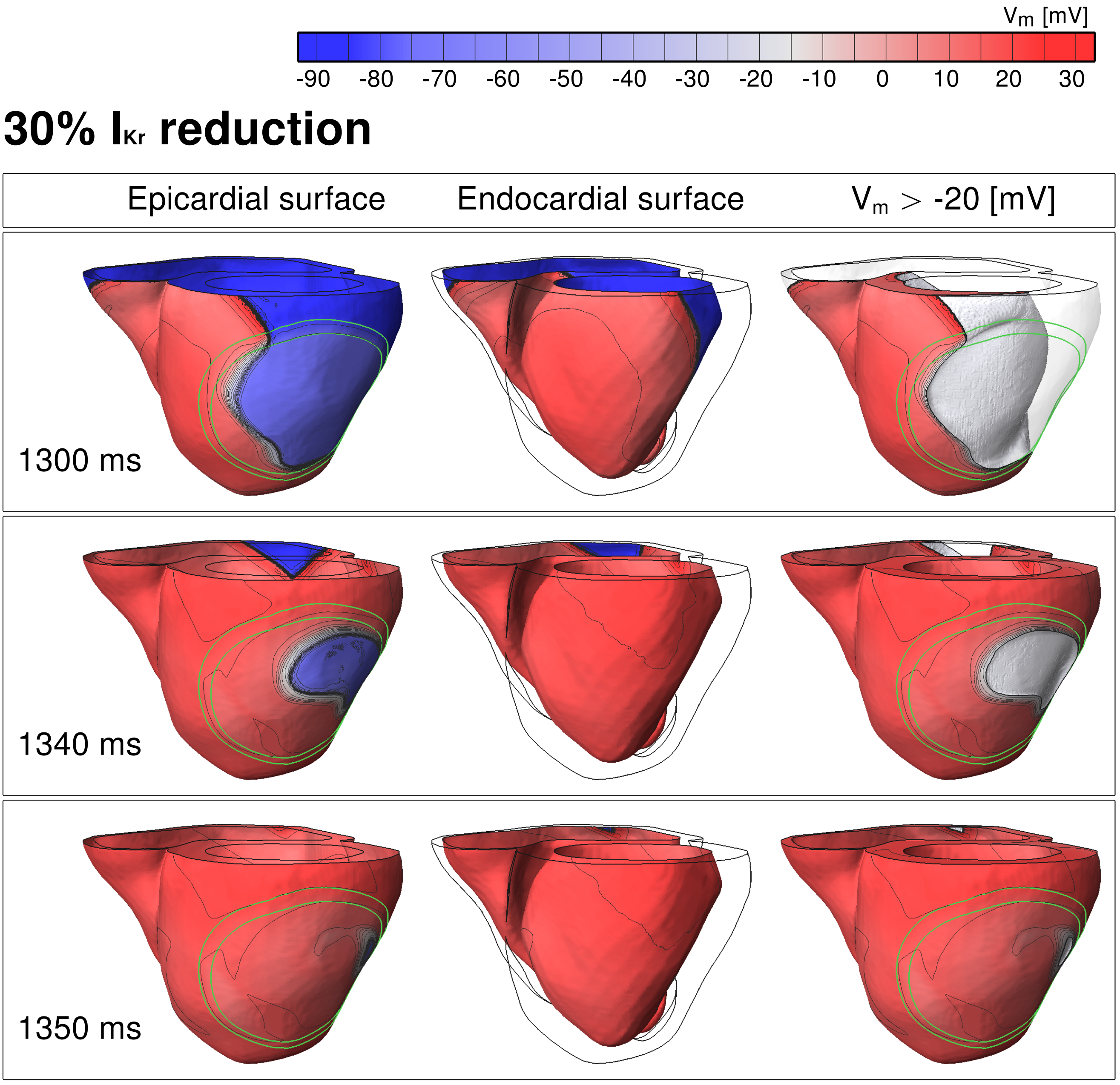

V_m [mV]
-90 -80 -70 -60 -50 -40 -30 -20 -10 0 10 20 30
30% I_{Kr} reduction
Epicardial surface
Endocardial surface
V_m > -20 [mV]
1300 ms
1340 ms
1350 ms

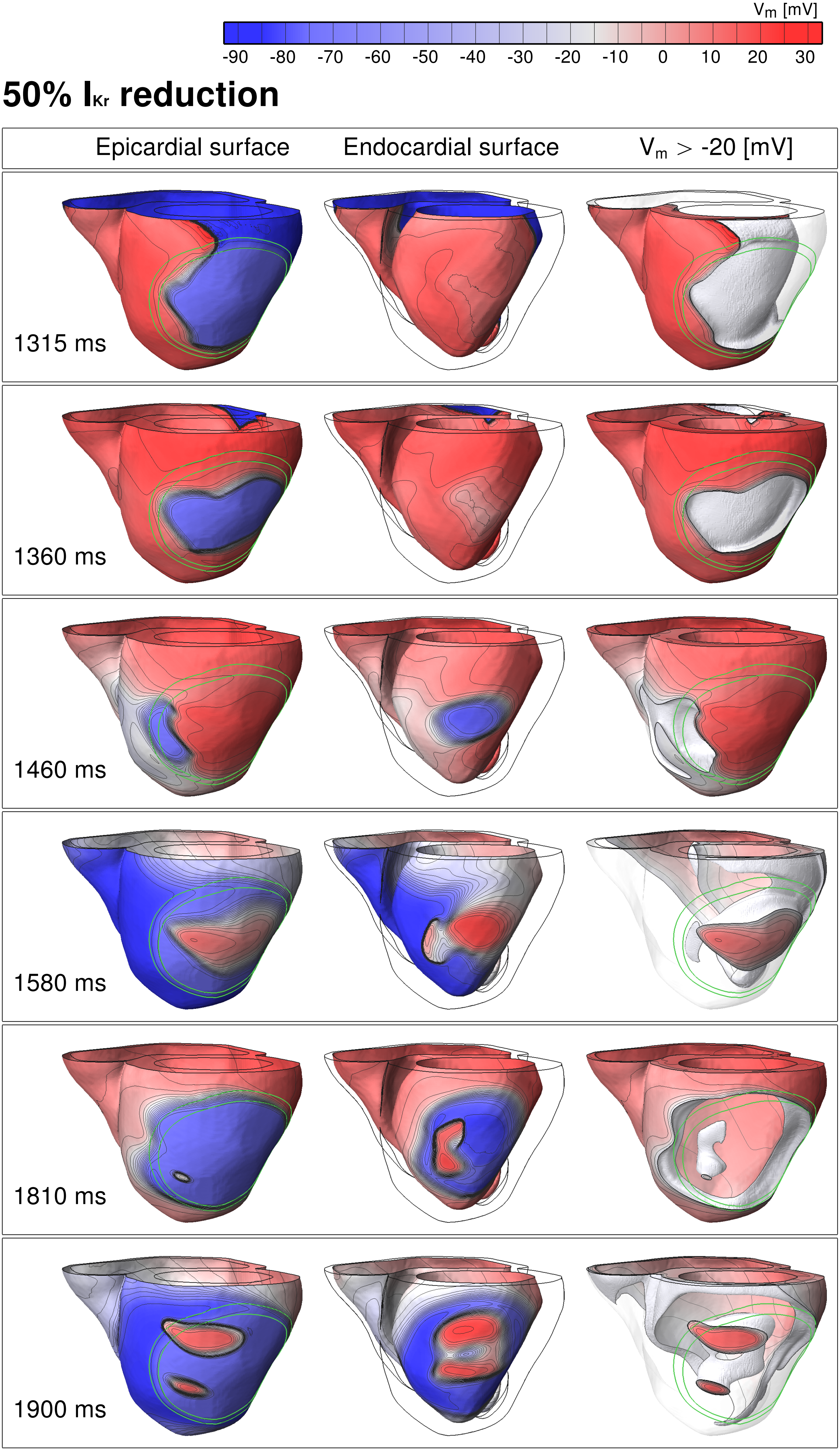
Vm [mV]
-90 -80 -70 -60 -50 -40 -30 -20 -10 0 10 20 30
50% IKr reduction
Epicardial surface
Endocardial surface
Vm > -20 [mV]
1315 ms
1360 ms
1460 ms
1580 ms
1810 ms
1900 ms
